# Allocryptopine, tetrahydropalmatine, and tetrahydroberberine N-oxide alkaloids alleviate cellular stress by modulating calcium homeostasis and the MAPK and akt/GSK-3β/tau signaling pathways

**DOI:** 10.3389/fphar.2025.1589390

**Published:** 2025-11-25

**Authors:** Serap Nigdelioglu Dolanbay, Belma Aslim

**Affiliations:** Department of Biology, Faculty of Science, Gazi University, Ankara, Türkiye

**Keywords:** Akt/GSK-3β/tau signaling pathway, *Glaucium grandiflorum*, intracellular Ca^2+^ homeostasis, MAPK signaling pathways, alkaloid

## Abstract

**Introduction:**

*Glaucium grandiflorum* Boiss. and A. Huet subsp. *refractum* (Papaveraceae) is a plant used in traditional medicine for analgesic, anti-inflammatory, sedative, and bronchitis treatment. Benzylisoquinoline derivative alkaloids in its content are responsible for its muscle relaxant, antitussive, and antioxidant effects. It attracts attention as a promising natural source, especially for neurodegenerative diseases (NDs) and conditions associated with oxidative stress, with its neuroprotective, antiproliferative, and calcium homeostasis regulating effects. This study investigates the neuroprotective effects of allocryptopine, tetrahydropalmatine, and tetrahydroberberine N-oxide rich alkaloid extract from *Glaucium grandiflorum* (GGAE).

**Methods:**

The plant was collected, identified, and the GGAE was prepared by macerating dried, pulverized material in chloroform. The GGAE’s neuroprotective properties were assessed using the rat pheochromocytoma (PC12) cell line. Intracellular calcium levels were analyzed via flow cytometry; gene expression of L-type voltage-gated calcium channel subtypes was evaluated with qRT-PCR; and the phosphorylation status of key proteins (p-ERK1/2, p-JNK, p-p38, p-Akt, p-GSK-3β, and p-Tau) was determined using Western blotting. The binding energies and contact residues of alkaloids (allocryptopine, tetrahydropalmatine, and tetrahydroberberine N-oxide) found in the GGAE were determined to target proteins (AKT1, CACNA1C, CACNA1D, ERK1/2, GSK3β, JNK, P38, and TAU).

**Results:**

The results suggest that the GGAE helps maintain intracellular calcium homeostasis and functions as an L-type Ca_2+_ channel blocker, crucial for neuronal survival. It modulates key signaling pathways by dephosphorylating stress-related proteins p-ERK1/2, p-JNK, and p-p38, while enhancing cell survival pathways by phosphorylating p-Akt (Ser 473) and p-GSK-3β (Ser 9). Additionally, the GGAE reduces pathological phosphorylation of p-Tau (Ser 396 and Thr 212), proteins associated with NDs. Molecular docking results demonstrated that alkaloids exhibit strong binding energies to target proteins.

**Discussion:**

These findings suggest that the GGAE exerts a comprehensive neuroprotective effect, positioning it as a promising therapeutic candidate for the treatment of NDs.

## 1 Introduction

Neurodegenerative diseases (NDs) are a group of diseases with a cluster of pathological conditions resulting from the loss of neurons and synapses in addition to slowly progressive and irreversible dysfunction in specific regions of the central nervous system (CNS) (Jellinger, 2010; Golpich et al., 2017; Wilson et al., 2023). NDs share a set of molecular pathologies, such as dysregulation of intracellular Ca^2+^ homeostasis, activation of mitogen-activated protein kinases (MAPK) (ERK1/2, JNK and p38) signaling pathways and dysregulation of Akt/GSK-3β/Tau neural death signaling pathway (Cui et al., 2021; Fricker et al., 2018; Joseph et al., 2020; Kim and Choi, 2010; Muddapu et al., 2020). In NDs, dysfunctions in ion-guided ATPases, glucose, and glutamate transporters cause the opening of Ca^2+^ channels on axons, and the opening of these channels causes excessive cellular Ca^2+^ influx (Anekonda and Quinn, 2011; Boczek et al., 2012). Excessive cellular Ca^2+^ load causes apoptotic neural death mediated by caspase activation (Berliocchi et al., 2005). It has been reported that dysfunctions in voltage-gated calcium channels (VGCCs) (The CACNA gene family, which encodes the alpha subunits of VGCC complexes), which are particularly expressed in the CNS, may play a critical role in NDs (Anekonda and Quinn, 2011; Nimmrich and Gross, 2012; Proft and Weiss, 2015; Szymanowicz et al., 2024). MAPKs are a group of serine/threonine kinase signaling molecules that are involved in a wide range of cellular functions. MAPKs are triggered by threonine and tyrosine phosphorylation by MAPK1 or MAPK/ERK kinases (MEKs). MAPK1 is then triggered by MAPK2 or MEK kinases (MEKKs), which respond to a variety of extracellular stimuli, including hydrogen peroxide (H_2_O_2_). Thus, the MAPK pathways comprise three key sequentially active protein kinases: MAPK2—MAPK1—p38-/ERK-/JNK-MAPK, and activation of these three protein kinases is linked to NDs (Ijomone et al., 2024). The activation of the phosphatidylinositol 3-kinase (PI3K)/Akt pathway enhances cell survival, including neurons in the central nervous system. In PI3K/Akt-inactivated metabolism, apoptotic regulator proteins like Bcl-2–associated X protein (Bax) and Bcl-2 may become imbalanced and activate caspase-3, resulting in neuronal death. Inactivation of PI3K/Akt activates glycogen synthase kinase-3β (GSK-3β), resulting in tau hyperphosphorylation, a major cause of NDs (Kim et al., 2024).

Currently, no NDs is curable and current treatments only control symptoms or halt disease progression (Durães et al., 2018). At the same time, the phenotypic heterogeneity among NDs patients, the lack of effective biomarkers, the limited translational potential of experimental models, and other factors hinder the development of drugs for NDs (Pasteuning-Vuhman et al., 2021). Therefore, studies are ongoing to identify various treatments that alleviate these diseases and to discover drugs that block the progression of NDs by targeting molecular pathways, with research particularly focused on multi-target therapeutic options. This focus is consistent for derivatives of natural products such as phytochemicals with multi-target properties (Efferth and Koch, 2011; Kumar and Khanum, 2012; Kurz and Perneczky, 2011). Phytochemicals are divided into various groups according to their chemical structures (Kennedy and Wightman, 2011). These natural product derivatives are alkaloids, phenols, steroids, glycosides, tannins, terpenoids, and phytoalexins (Koche et al., 2016).

*Glaucium grandiflorum* Boiss. and A. Huet subsp. refractum (Nábělek) Mory is a plant that belongs to the Papaveraceae family, used in traditional medicine for the treatment of various diseases (Kupeli et al., 2003). This plant is widely used by the folk, especially in the Mediterranean and Middle East regions, for its analgesic, anti-inflammatory, sedative, and anti-bronchitis effects (Toker et al., 2004). Benzylisoquinoline derivative alkaloids contained in it are known as the compounds responsible for its pharmacological activities (Shams et al., 2015). Its traditional uses make use of its muscle relaxant, antitussive, and antioxidant effects (Zarghami Moghadam et al., 2018). The confirmation of neuroprotective, antiproliferative, and calcium homeostasis regulating effects of *G. grandiflorum* in modern pharmacological studies has created an increasing interest in further examining the biologically active compounds of this plant, especially its alkaloids (Sezgin ve Sahin, 2020).

Alkaloids have anti-apoptotic, anti-autophagic, anti-genotoxic, anti-inflammatory, anti-mutagenic, anti-oxidant, Ca^+2^ homeostasis regulator, and neurotransmitter modulator properties, among others. Due to its multi-target properties, it has the potential to treat NDs by targeting mechanisms such as dysregulation in Ca^2+^ homeostasis, activation of MAPK signaling pathways, protein misfolding/aggregation, and reduced neural survival (Fan et al., 2019; Girdhar et al., 2015; Hussain et al., 2018; Hügel et al., 2021; Michel et al., 2016; Moura et al., 2007; Nie et al., 2016; Xie et al., 2013; Yang et al., 2021; Zhou et al., 2020; Zhou and Zhou, 2012).

GC-MS, LC-MS/MS, and ^1^H and ^13^C NMR analyses were previously employed to identify the major alkaloids -allocryptopine, tetrahydropalmatine, and tetrahydroberberine N-oxide (trans-cannadine-N-oxide)- in the alkaloid extract from *G. grandiflorum* (GGAE) (Nigdelioğlu Dolanbay et al., 2021; Nigdelioğlu Dolanbay et al., 2023). Earlier findings demonstrated that GGAE suppressed H_2_O_2_-induced neuronal apoptosis, potentially through inhibition of the mitochondrial apoptotic pathway and regulation of the cell cycle in PC12 cells (Nigdelioğlu Dolanbay et al., 2021). Additionally, GGAE was shown to attenuate lipopolysaccharide (LPS)-induced inflammation by downregulating pro-inflammatory cytokines and mediators, as well as p38 MAPK signaling in BV2 cells (Nigdelioglu Dolanbay et al., 2023). The extract also mitigated H_2_O_2_-induced oxidative stress, likely by modulating the NRF2-KEAP1 pathway in PC12 cells (Nigdelioğlu Dolanbay et al., 2023).

In this article, experimental studies were conducted to discover the effects of plant-derived alkaloids with neuroprotective effects on the common mechanisms of NDs. In these experimental studies, the effects of GGAE obtained with chloroform solvent on dysregulation of intracellular Ca^2+^ homeostasis, activation of MAPK (ERK1/2, JNK and p38) signaling pathways, and dysregulation of Akt/GSK-3β/Tau neural death signaling pathway were investigated for the first time.

## 2 Materials and methods

### 2.1 Plant used in the study

*Glaucium grandiflorum* Boiss. and A. Huet subsp. *refractum* (Nábělek) Mory, used in this study, was collected from Beypazarı (Ankara, Turkey) by Prof. Dr. Zeki Aytaç on 27 July 2015. The plant material was identified and taxonomically authenticated based on morphological characteristics using standard floras, by Prof. Dr. Zeki Aytaç, a specialist in the field of plant taxonomy. A voucher specimen (Herbarium No: ZA10700) was deposited at the Gazi University Herbarium. The collection site is located at the coordinates 40°1′53″N, 32°16’18”E (the 10th kilometer of the Beypazarı–Ankara road, from gypsum-rich soil areas). The aerial parts of the authenticated plant were dried and ground into powder for use in experimental studies.

### 2.2 PC12 cell line used in the study

The cells used in the study are the pheochromocytoma-12 (PC12, ATCC^®^ CRL-1721™) cell line obtained from *Rattus norvegicus* and derived from rat adrenal medulla. PC12 cells were grown in Dulbecco’s modified Eagle’s medium (DMEM) (41966029-Gibco, USA) supplemented with 10% heat-inactivated fetal bovine serum (10270106-Gibco, USA), 10% heat-inactivated horse serum (26050088-Gibco, USA), 1% penicillin/streptomycin (P4333-Sigma-Aldrich, Germany) and 1% L-Glutamine (A2916801-Gibco, USA) at 37 °C in an incubator (MCO-18AC-PE-Panasonic, Japan) containing 5% CO_2_ and providing a humidified atmosphere. The growth of PC12 cells grown in flasks (3290-Corning, England) coated with collagen (A1048301-Thermo Fisher Scientific, USA) was monitored by changing the medium every 2 days. PC12 cells that showed 80%–90% spread in the flasks were removed, counted, and transferred to 96- and 6-well microplates at a density appropriate for experimental requirements (10^4^, 10^5^ or 5 × 10^5^ cells/well). Differentiation of PC12 cells into neuron-like cells was achieved after 4 days of incubation with 100 ng/mL nerve growth factor (NGF) (G5141-Promega, USA).

### 2.3 Preparation of GGAE

Ten grams of powdered plant samples were extracted with 150 mL of chloroform solvent in a Soxhlet apparatus (LabHeat, Germany) for 8 h. The solvents were removed by a temperature-controlled (40 °C) and low-pressure rotary evaporator (Heidolph Laborota 4000, Germany). The plant extracts were dissolved in 10 mL of 2% sulfuric acid and treated with 3 × 50 mL of diethyl ether ((C_2_H_5_)_2_O). The aqueous acid solution was adjusted to pH 9.0 with ammonium hydroxide (NH_4_OH), and 3 × 50 mL of chloroform was added thereafter. The solvents of the samples dried with sodium sulfate (Na_2_SO_4_) were evaporated in a temperature-controlled (40 °C) and low-pressure rotary evaporator. The obtained GGAE was stored at +4 °C until further use in experimental studies.

### 2.4 Neurodegenerative cell model and different treatment groups

According to the results obtained from the preliminary dose- and time determination studies conducted with MTT in our previous studies, the PC12 cells were grouped as follows (Nigdelioglu Dolanbay et al., 2021):

Control group: PC12 cells were grown in DMEM containing NGF for 6 days (The proliferation medium was replaced with differentiation medium containing NGF every 2 days). The NGF in the medium triggers neuronal differentiation in PC12 cells, leading to morphological changes and the acquisition of neuron-like characteristics; these cells are referred to as fPC12 due to their differentiated phenotype.

H_2_O_2_ group (neurodegenerative cell model): H_2_O_2_ was dissolved in DMEM containing NGF to a final concentration of 200 μM. fPC12 cells were treated with H_2_O_2_ for 24 h on the sixth day.

GGAE group: GGAE was dissolved in DMEM containing NGF to a final concentration of 100–500 μg/mL. After 4 days of treatment with differentiation medium, fPC12 cells were treated with alkaloid extracts for 18 h on the fifth day.

GGAE + H_2_O_2_ group (experimental group): fPC12 cells were treated with GGAE (100–500 μg/mL) for 18 h on the fifth day and then treated with 200 μM H_2_O_2_ for 24 h on the sixth day.

### 2.5 Analysis of intracellular calcium levels by flow cytometry

For flow cytometric analyses, 6-well microplates were coated with collagen and then PC12 cells were seeded with 1 × 10^5^ cells per well. After 4 days of NGF treatment, fPC12 cells were incubated with GGAE (100, 250, and 500 μg/mL) dissolved in 2 mL of medium (containing 100 ng/mL NGF) in a 5% CO_2_ incubator at 37 °C for 18 h. Then, the cells were incubated in NGF medium containing 200 µM H_2_O_2_ under the same conditions for 24 h. Cells grown in normal NGF cell culture conditions without H_2_O_2_ and GGAE treatment were used as controls. Treated and untreated cells were collected and centrifuged at 1,000 rpm for 10 min. After removing the supernatant, cells were suspended with Ca^2+^-free phosphate-buffered saline (PBS) and incubated with 5 µM eBioscience™ Calcium Sensor Dye eFluor™ 514 (65-0859-70-InvitrogenTM, USA) in the dark at 37 °C for 30 min and at room temperature for another 15 min. After incubation, cells were washed twice with PBS to minimize background fluorescence and nonspecific staining. eFluor 514 fluorescence intensity was measured by excitation at 490 nm and emission at 514 nm. Analysis results are given as eFluor 514 fluorescence intensities expressed as fold change compared to the control group. Flow cytometry analyses were performed using a NovoCyte^®^ 3000 Flow Cytometry device (ACEA Biosciences, Inc., USA) and a NovoCyte^®^ 2060R Flow Cytometry device (Agilent, USA). The samples were analyzed using the ACEA NovoExpress software. All flow cytometry analyses were performed as a service procurement at Bilkent University THORVACS Biotechnology Laboratory.

### 2.6 Analysis of gene expression levels of L-type voltage-gated calcium channel subtypes by qRT-PCR

Total RNA isolation from fPC12 cells was performed using a RNeasy Mini kit (74104-Qiagen, USA), a Quick-RNA™ MiniPrep kit (R1054-Zymo Research, USA), and a PureLink™ RNA Mini kit (12183018A-InvitrogenTM, USA), which were used according to the manufacturer’s instructions. Complementary DNA (cDNA) synthesis from RNA samples was performed using a SensiFAST cDNA Synthesis kit (65053-Bioline, USA) and a High-Capacity cDNA Reverse Transcription kit (4368814-Thermo Fisher Scientific, USA), which were used according to the manufacturer’s instructions. qRT-PCR reaction was performed using cDNAs and specific primers were used as templates (Supplementary Table 1). In the study, a SensiFASTTM SYBR^®^ Lo-ROX kit (94005-Bioline, USA) and an AMPIGENE^®^ qPCR Green Mix Lo-ROX kit (ENZ-NUC103-1000-Enzo, Switzerland) were used. All cDNA samples in the experiments were run on an Applied Biosystems™ QuantStudioTM 3 Real-Time PCR device (A28131-Thermo Fisher Scientific, USA). Twenty μL qRT-PCR reaction was performed after 2 min of polymerase activation at 95 °C, one cycle of 5-s denaturation at 95 °C, 40 cycles of 10–30-s annealing at 60 °C–65 °C, and 20-s extension at 72 °C. In the study, glyceraldehyde-3-phosphate dehydrogenase (GAPDH), a housekeeping gene, was used as an internal control and in the evaluation of the results; Cav_1.2_ and Cav_1.3_ were normalized by ratio to GAPDH, and then mRNA expression levels were compared to the control. For these analyses, the comparative cycle threshold (CT) method (2^−ΔΔCT^ method) was used.

### 2.7 Western blot hybridization technique for protein expression studies

For total protein isolation, treated and untreated cells were washed with cold 1 × PBS and collected in a sterile tube. The tubes were centrifuged for 5 min at 2,500 × g at +4 °C. A protease inhibitor cocktail tablet (11 836 153 001-Roche, Germany or A32963-Thermo Fisher Scientific, USA) and a phosphatase inhibitor cocktail tablet (04 906 837 001-Roche, Germany or A32957-Thermo Fisher Scientific, USA) were added to the cell pellets and 500 µL of a cold radioimmunoprecipitation (Ripa) lysis buffer (89900-Thermo Fisher Scientific, USA) was applied. The tubes were incubated for 30 min at +4 °C by vortexing every 5 min. Then, the tubes were centrifuged for 30 min at 16,000 × g at +4 °C, and the protein amounts in the supernatant were determined. Protein amounts were determined using a Pierce Bicinchoninic acid (BCA) Protein Assay kit (23228-Thermo Fisher Scientific, USA) in accordance with the manufacturer’s instructions. To create the standard curve, 8 Pierce Bovine Serum Albumin (BSA) (23209-Thermo Fisher Scientific, USA) dilutions with concentrations ranging from 25 to 2000 μg/mL were created. The absorbance values ​​of 25–2000 μg/mL standard BSA solutions and the samples at 562 nm were measured. The total protein amount contained in each sample was calculated as μg/mL with the obtained standard graph. For the Trans-Blot Turbo Transfer System (Bio-Rad, USA), protein samples were mixed at 10–30 μg per well with 2 × loading buffer (Laemmli Sample Buffer) (1610737-Bio-Rad, USA) containing 5% β-mercaptoethanol at a volume of 1:1 and denatured at 90 °C for 10 min. The 4%–20% ready-made bis-tris gel (Mini-PROTEAN^®^ TGX™ Precast Protein Gels, 10-well, 50 μL) (4561094-Bio-Rad, USA) used in the Trans-Blot Turbo Transfer System was placed in the vertical electrophoresis tank for sodium-dodecyl-sulfate polyacrylamide gel electrophoresis (SDS-PAGE). After filling the tank with 1 × Tris/Glycine/SDS (1610732- Bio-Rad, USA) running buffer, protein samples and markers were loaded into the wells of the gel. In the studies, PageRuler™ Plus Prestained Protein Ladder, 10–250 kDa (26619-Thermo Fisher Scientific, USA) was used as a marker for the analysis of the Cav_1.2_ and Cav_1.3_ proteins, while the PageRuler™ Prestained Protein Ladder (10–170 kDa) (26616-Thermo Fisher Scientific, USA) was used for the analysis of other proteins. The running process was carried out at 120 V for 60 min (Mini Trans-Blot®Cell-Bio-Rad, USA). A Trans-Blot Turbo RTA Mini 0.20 µm PVDF Transfer kit for 40 blots (1704272-Bio-Rad, USA) was used to transfer proteins from the SDS gel to the polyvinylidene fluoride (PVDF) membrane. Then, the proteins were transferred from the gel to the PVDF membrane using the Trans-Blot Turbo Transfer System gel transfer device (1704150-Bio-Rad, USA). After the transfer process, the PVDF membrane was incubated in a blocking solution consisting of 1 × Tris-buffered saline (TBS) (1706435-Bio-Rad, USA) containing 5% milk powder and 0.1% tween-20 for 1 h on a shaker. Then, the membrane was washed 3 times for 5 min each with 5 mL of 1 × TBS containing 0.1% tween-20. To demonstrate the proteins expressed in cells, primary antibodies [CACNA1C and CACNA1D antibodies target subunits of L-type voltage-gated calcium channels involved in neuronal calcium influx] were used. Total and phosphorylated forms of ERK1/2, JNK, p38, and AKT1 were analyzed to evaluate pathways related to cell proliferation, stress response, and survival. GSK-3β activation and its regulation by AKT were examined using both total and phospho-GSK-3β antibodies. Additionally, phosphorylation levels of the tau protein were assessed using site-specific phospho-tau antibodies (Ser396 and Thr212) alongside total tau detection (TAU-5)] were prepared by diluting them in 10 mL of blocking solution according to the manufacturer’s instructions. The PVDF membrane that was first blocked and then washed was treated with the primary antibody for 2 h at room conditions. Then, the membrane was washed with 5 mL of 0.1% tween-20 containing 1 × TBS 3 times for 5 min each. Similar to the primary antibody, the secondary antibody was also prepared by diluting them in 10 mL of blocking solution according to the manufacturer’s instructions. After primary antibody was washed, the PVDF membrane was treated with the secondary antibody for 1 h at room conditions (Supplementary Table 2). Then, the membrane was washed with 5 mL of 0.1% tween-20 containing 1 × TBS 3 times for 5 min each. After the washing step, the PVDF membrane was treated with 10 mL of alkaline phosphatase (AP) conjugate substrate kit (1706432-Bio-Rad, USA) for 1 h on a shaker. Then, the membrane was washed for one or 2 times for 10 min each in 10 mL of sterile distilled water (dH_2_O) to remove excess dye. Protein bands on the membrane were detected in the gel and blot imaging system (Gel DocTM XR + Imaging System-Bio-Rad, USA). Analysis of protein bands was performed using the Bio-Rad Image Lab 6.0.1 program.

### 2.8 Determination of binding energies and contact residues of ligands to target proteins via molecular docking

Three alkaloids (allocryptopine (major), tetrahydropalmatine, and tetrahydroberberine N-oxide) found in the GGAE were downloaded from PubChem as ligands in the sdf format. Target proteins (CACNA1C, CACNA1D, ERK1/2, JNK, P38, AKT1, GSK3β, and TAU) were downloaded from the RCDB PDB protein data bank (https://www.rcsb.org/) in the pdb format. Blind docking of ligands and target proteins under cavity detection guidance was performed with the CB-DOCK2 (https://cadd.labshare.cn/cb-dock2/index.php).

### 2.9 Statistical analysis

In the statistical analyses of the study data, IBM SPSS version 21.0 was used. In all experiments, statistical differences between the untreated (control) group and the H_2_O_2_-treated group were determined by the Levene Independent Sample T-test. Multiple group comparisons with respect to concentration and/or time-related differences between the H_2_O_2_-treated group and the experimental groups were carried out using One-way analysis of variance (ANOVA) and then post hoc Tukey HSD test. The analyses were performed repeatedly and in parallel, and the results were presented as mean value ±standard deviation (SD). Statistical difference was determined at *p* < 0.05 at a significance level of 95% or *p* < 0.01 at a significance level of 99%.

## 3 Results

### 3.1 Suppressive effect of GGAE on intracellular calcium level

**FIGURE 1:**
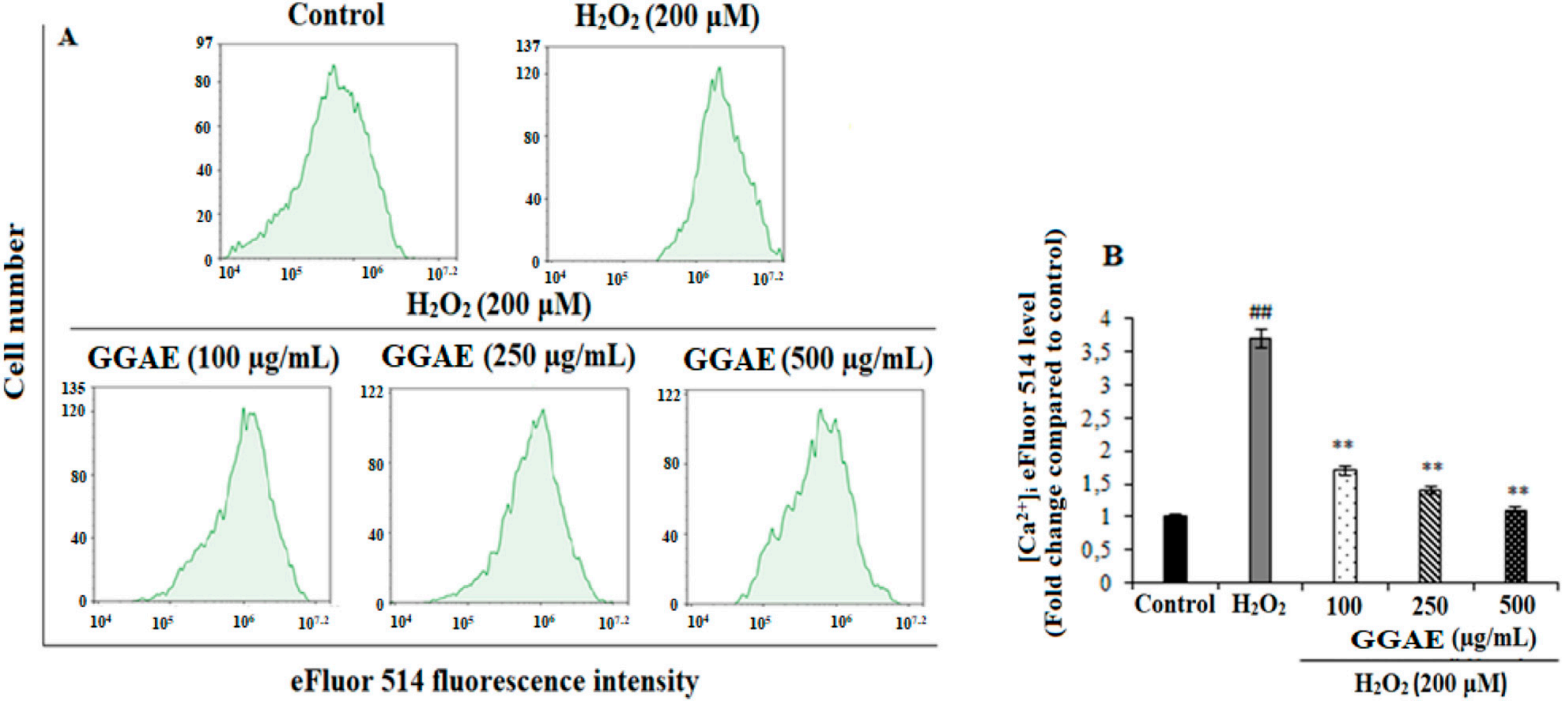
Determination of the suppressive effect of GGAE on H_2_O_2_-induced intracellular Ca^2+^ level by flow cytometry analysis **(A)** Flow cytometry histogram graph. **(B)** Bar graph (Quantitative analysis of [Ca^2+^]_i_ based on eFluor 514 fluorescence intensities expressed as fold change compared to the control group). Mean value ±SD, n: 3. ^##^ *p* < 0.01 [Ca^2+^]_i_ in H_2_O_2_-treated compared to control; ^**^ *p* < 0.01 [Ca^2+^]_i_ in experimental groups compared to H_2_O_2_-treated group.

Our results showed that [Ca^+2^]_i_ increased 3.7-fold in H_2_O_2_-treated cells compared to the control group (^##^ *p* < 0.01). On the other hand, the treatment of GGAE regulated Ca^2+^ homeostasis in fPC12 cells by suppressing the increased [Ca^2+^]_i_, with the best result being obtained with the 500 μg/mL treatment (3.4-fold compared with the H_2_O_2_-treated group) (^**^ *p* < 0.01) (Figure 1).

### 3.2 Suppressive effect of GGAE on L-type voltage-gated calcium channel subtypes at the level of gene and protein expression

**FIGURE 2:**
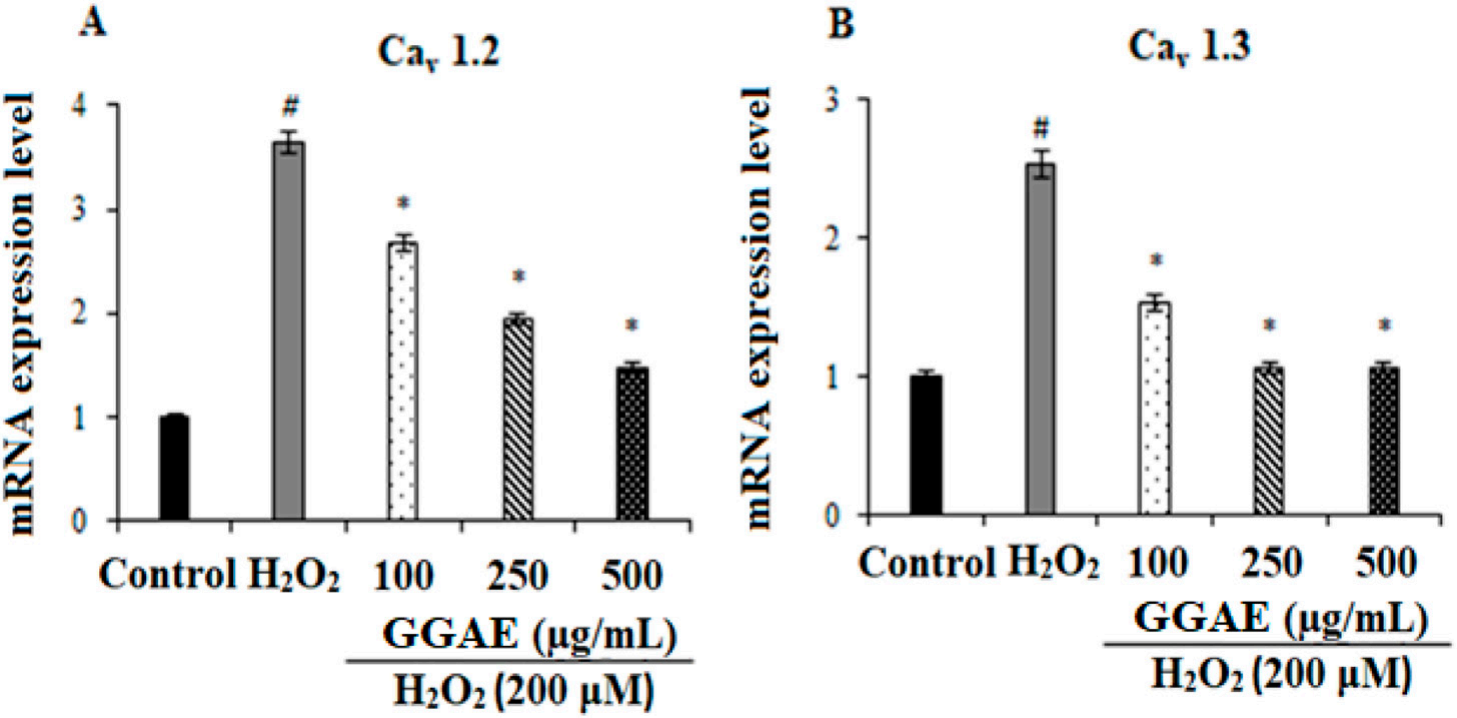
Effect of GGAE on the expression levels of Cav_1.2_ **(A)** and Cav_1.3_ **(B)** genes in fPC12 cells in which L-type VGCC subtypes were activated by H_2_O_2_. Mean value ±SD, n:3. ^#^ *p* < 0.05 mRNA expression level in H_2_O_2_-treated group compared to control; ^*^ *p* < 0.05 mRNA expression level in experimental groups compared to H_2_O_2_-treated group.

**FIGURE 3:**
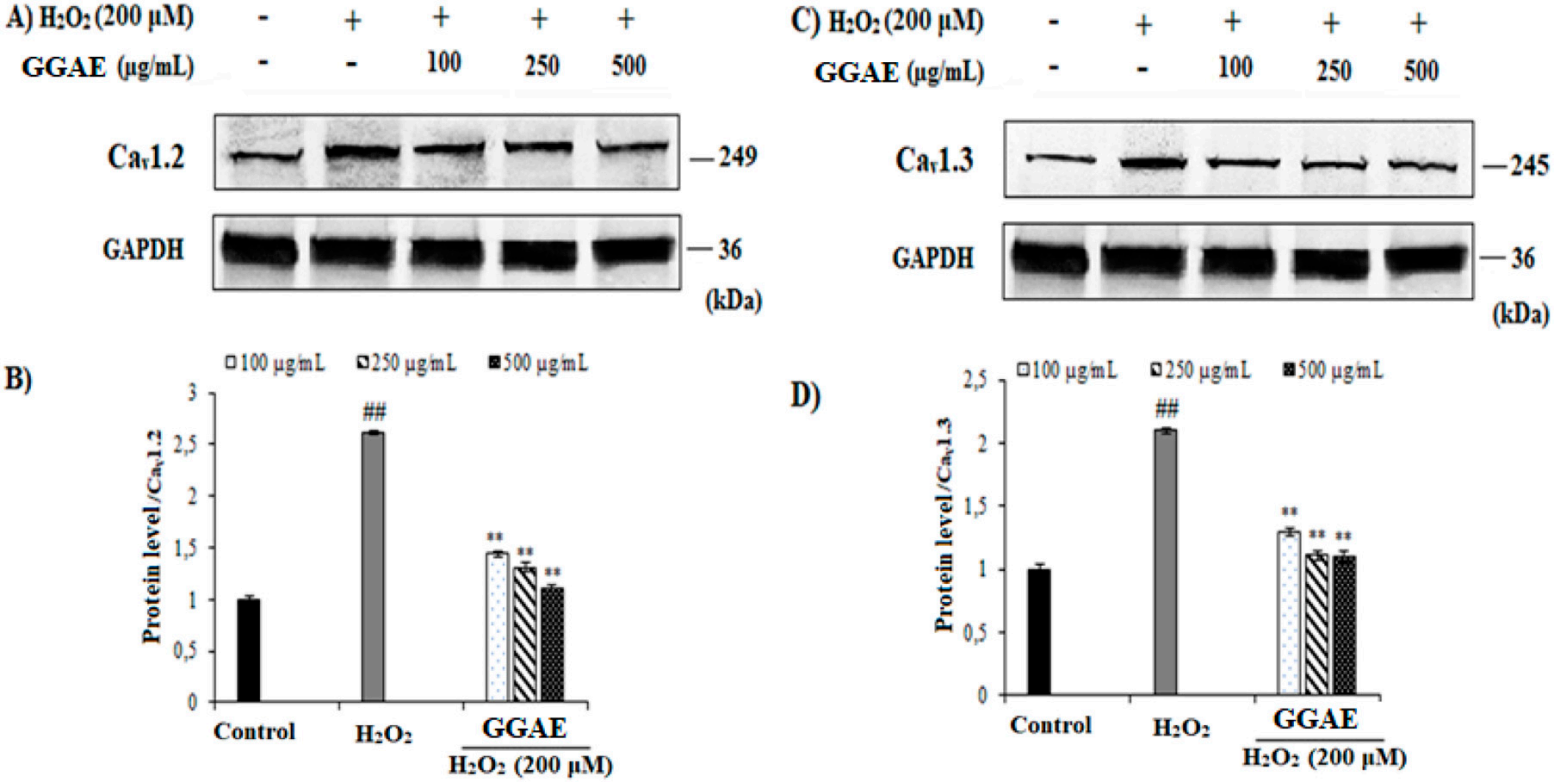
Effect of GGAE on the expression levels of Cav_1.2_ **(A,B)** and Cav_1.3_ **(C,D)** proteins in fPC12 cells in which L-type VGCC subtypes were activated by H_2_O_2_. Mean value ±SD, n:2. ^##^ *p* < 0.01 Cav_1.2_ and Cav_1.3_ protein levels in the H_2_O_2_-treated group compared to the control; ^**^ *p* < 0.01 Cav_1.2_ and Cav_1.3_ protein levels in the experimental groups compared to the H_2_O_2_-treated group.

Our results showed that, in H_2_O_2_-treated cells, Cav_1.2_ and Cav_1.3_ were increased at the gene (3.6- and 2.5-fold, respectively) and protein levels (2.61- and 2.10-fold, respectively) compared with the control group (^#^ *p* < 0.05). On the other hand, the treatment of GGAE suppressed Cav_1.2_ and Cav_1.3_ at the gene (2.5 and 2.4-fold compared with the H_2_O_2_-treated group, respectively) and protein levels (2.4 and 1.9-fold compared with the H_2_O_2_-treated group, respectively), with the best results being obtained with the treatment of 500 μg/mL for Cav_1.2_ and 250 μg/mL for Cav_1.3_ (^*^ *p* < 0.05) (Figures 2, 3).

### 3.3 Suppressive effect of GGAE on MAPK signaling pathways activation (ERK1/2, JNK, and p38) at the level of protein expression

**FIGURE 4:**
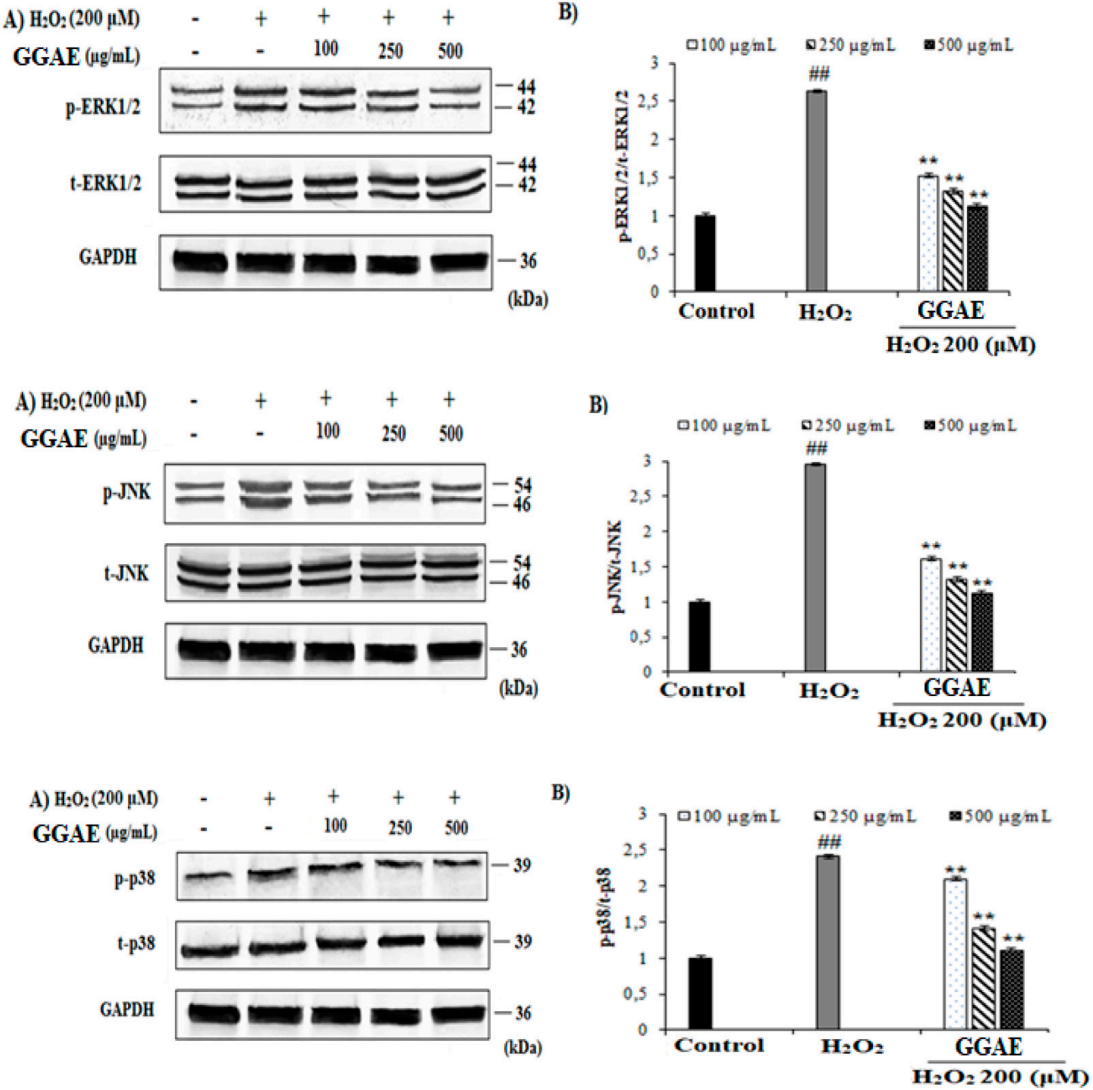
Determination of the suppressive effect of GGAE on H_2_O_2_-induced p-ERK1/2 (Thr 202/Tyr 204), p-JNK (Thr 183/Tyr 185), and p-p38 (Thr 180/Tyr 182) protein level by Western blot analysis. **(A)** Western blot image of p-ERK1/2, t-ERK1/2, p-JNK, t-JNK, p-p38, t-p38 and GAPDH. **(B)** Graph of densitometry values ​​showing changes in p-ERK1/2/t-ERK1/2, p-JNK/t-JNK, and p-p38/t-p38 ratio compared to control. Mean value ±SD, n:2. ^##^ *p* < 0.01 p-ERK1/2, p-JNK, and p-p38 protein level in the H_2_O_2_-treated group compared to control. ^**^ *p* < 0.01 p-ERK1/2, p-JNK, and p-p38 protein level in the experimental groups compared to the H_2_O_2_-treated group.

Our results showed that p-ERK1/2, p-JNK, and p-p38 MAPK activation levels in H_2_O_2_-treated cells were increased by 2.64, 2.96, and 2.41 folds, respectively, compared with the control group (^#^ *p* < 0.05). On the other hand, the treatment of GGAE suppressed the activation of the MAPK signaling pathway, and the best result was obtained from the treatment of 500 μg/mL (2.4, 2.7, and 2.2 folds, respectively, compared with the H_2_O_2_-treated group) (^*^ *p* < 0.05) (Figure 4).

### 3.4 Suppressive effect of GGAE on Akt/GSK-3β/tau neuronal death signaling pathway dysregulation at the level of protein expression

**FIGURE 5:**
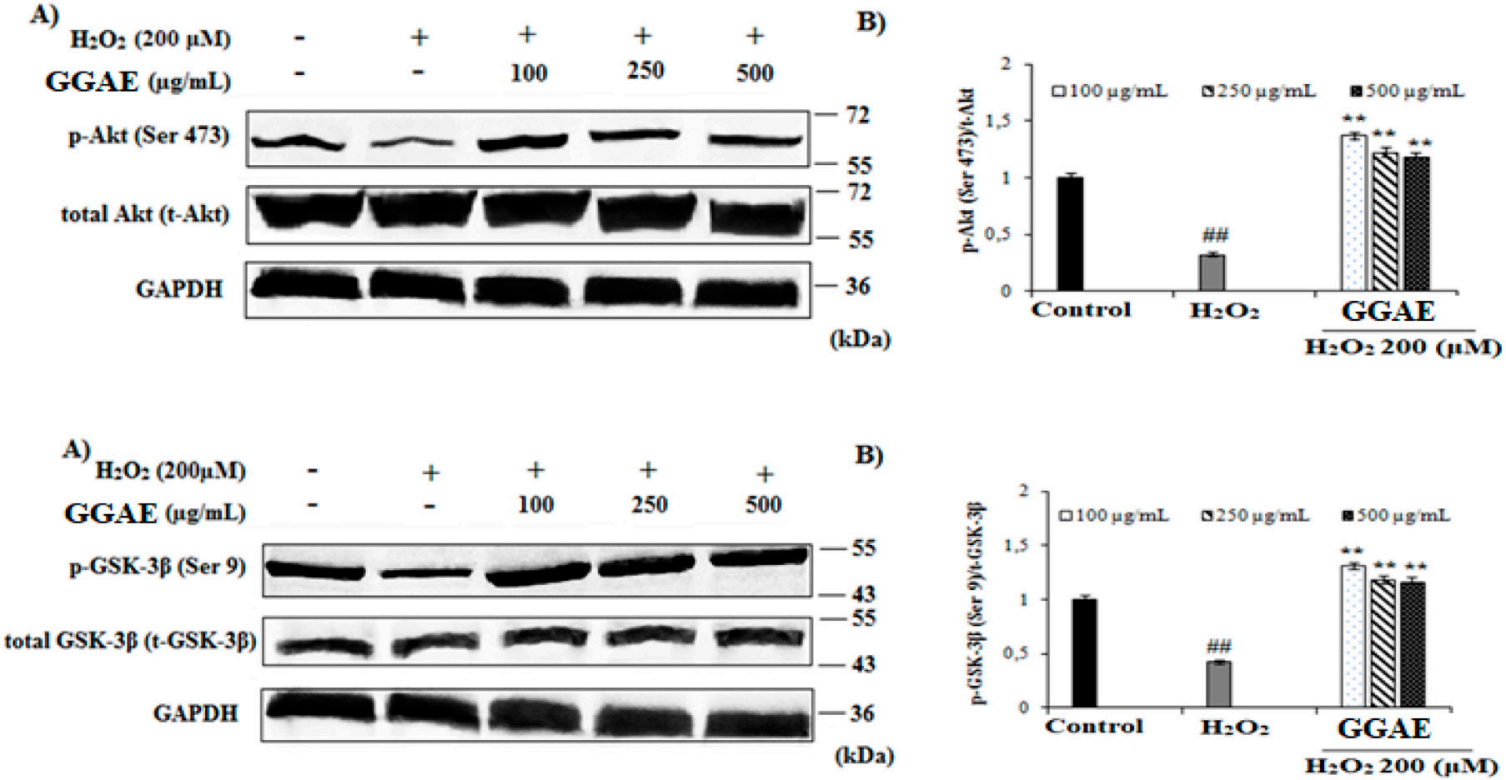
Determination of the activating effect of GGAE on H_2_O_2_-induced p-Akt (Ser 473) and p-GSK-3β (Ser 9) protein level by Western blot analysis. **(A)** Western blot image of p-Akt, t-Akt, p-GSK-3β, t-GSK-3β and GAPDH. **(B)** Graph of densitometer values ​​showing the changes in p-Akt/t-Akt and p-GSK-3β/t-GSK-3β ratio compared to control. Mean value ±SD, n:2. ^##^ *p* < 0.01 p-Akt and p-GSK-3β protein level in the H_2_O_2_-treated group compared to control. ^**^ *p* < 0.01 p-Akt and p-GSK-3β protein level in experimental groups compared to the H_2_O_2_ treated group.

Our results demonstrated that treatment with H_2_O_2_ significantly suppressed the protein levels of p-Akt and p-GSK-3β to 0.31 and 0.42 relative to control, respectively (#*p* < 0.05) The decrease in p-Akt and p-GSK-3β protein levels caused by H_2_O_2_ was reversed and increased with the treatment of GGAE, with the best result being obtained with the 100 μg/mL treatment (4.5 and 3.2-folds, respectively, compared with the H_2_O_2_-treated group) (^*^ *p* < 0.05) (Figure 5).

**FIGURE 6:**
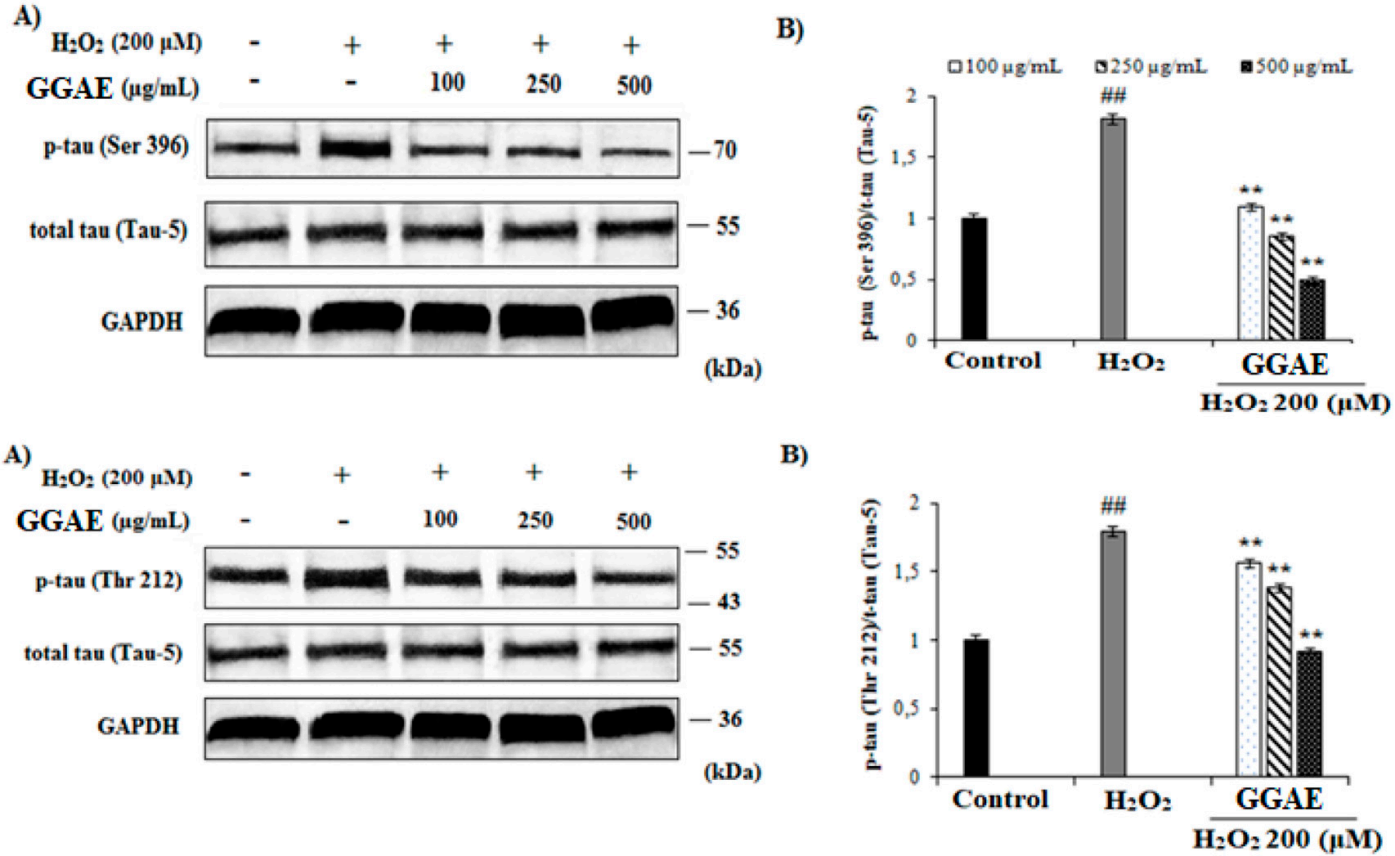
Determination of the suppressive effect of GGAE on H_2_O_2_-induced p-Tau (Ser 396) and p-Tau (Thr 212) protein level by Western blot analysis. **(A)** Western blot images of p-Tau, t-Tau and GAPDH. **(B)** Graph of densitometer values ​​showing changes in p-Tau (Ser 396)/t-Tau (Tau-5) and p-Tau (Thr 212)/t-Tau (Tau-5) ratio compared to control. Mean value ±SD, n:2. ^##^ *p* < 0.01 p-Tau protein level in the H_2_O_2_-treated group compared to control. ^**^ *p* < 0.01 p-Tau protein level in experimental groups compared to H_2_O_2_-treated group.

Our results showed that, in H_2_O_2_-treated cells, p-Tau (Ser 396) and p-Tau (Thr 212) protein levels increased 1.85 and 1.83 folds, respectively, compared with the control (^#^ *p* < 0.05). The increase in the level of p-Tau proteins formed by H_2_O_2_ was reversed and suppressed by the treatment of GGAE, with the best result being obtained with the 500 μg/mL treatment for both proteins (3.7-fold/p-Tau (Ser 396) and 2.0-fold/p-Tau (Thr 212), respectively, compared with the H_2_O_2_-treated group) (*p < 0.05) (Figure 6).

### 3.5 Binding energies and contact residues of ligands to target proteins via molecular docking

Binding energies (vina scores) and contact residues of ligands to target proteins are shown on Table 1. The order of binding energy (vina scores) of target proteins to ligands were as follows: JNK > AKT1 > ERK1/2 > CACNA1C > CACNA1D > GSK-3β > P38 > TAU. The order of binding energy (vina scores) of the ligands to target proteins were as follows: Allocryptopine > tetrahydropalmatine > tetrahydroberberine N-oxide. The molecular docking results demonstrated that the ligands exhibit strong binding energies to target proteins.

**TABLE 1:** Binding energies (vina score) and contact residues of ligands to target proteins.

Ligands	Target proteins	Vina scores	Contact residues	
Allocryptopine	CACNA1C	−8.4	Chain A: LYS427 GLN428 GLU431 GLU432 LYS435 VAL585 SER586 LEU587 PHE588 ARG590 PHE591 ASP592 VAL622 ARG623 LEU625 ARG626 LYS629 TRP634 ASN635 LEU637 SER638 LEU640 VAL641 ALA642 LEU644 LEU645 ASN646 VAL1055 LEU1059 ILE1172 PHE1176 MET1177 ILE1180	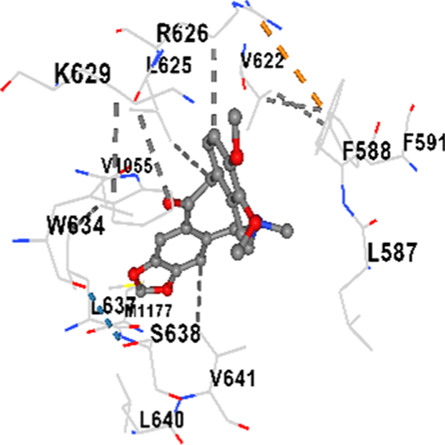
	CACNA1D	−8.0	Chain D: PHE288 VAL308 GLN309 ASN311 VAL312 ARG313 ASN314 SER350 TYR520 HIS524 PRO525 ASN526 GLN528 ILE532 THR558 LEU559 ASP560 LEU562 ASP563 ALA564 GLU565 LEU566 GLU567 ASP569 VAL572 ARG575 ASN576 VAL592 LYS593 SER594 GLU597 ASN895 LYS896 SER897 TYR898 TYR900 PRO1041 ASN1042 PRO1043 ASP1045 MET1046 VAL1047 GLN1049 PRO1050 ARG1051 TYR1052 ARG1053	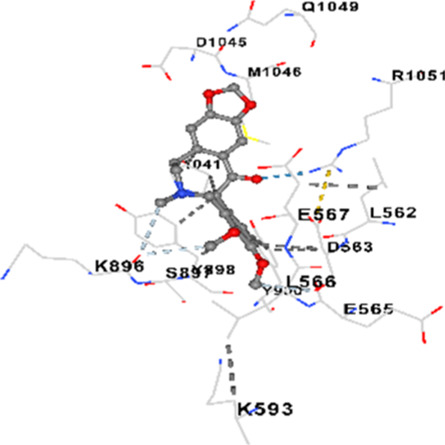
	ERK1/2	−8.8	Chain A: SER29 TYR30 ILE31 GLY32 GLU33 GLY34 ALA35 TYR36 GLY37 MET38 VAL39 CYS40 SER41 ALA52 LYS54 ILE56 ARG67 GLU71 LEU75 ILE84 GLN105 ASP106 LEU107 MET108 GLU109 THR110 ASP111 TYR113 LYS114 LYS151 SER153 ASN154 LEU156 CYS166 ASP167 GLY169 LEU170	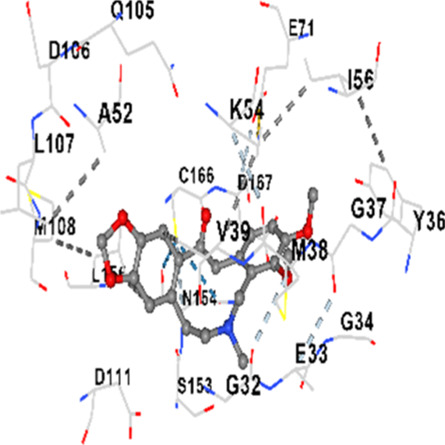
	JNK	−9.1	Chain B: LYS30 ILE32 GLY33 SER34 GLY35 ALA36 VAL40 CYS41 ALA42 VAL52 ALA53 LYS55 ILE86 MET108 GLU109 LEU110 MET111 ASP112 ALA113 ASN114 GLN117 LYS153 SER155 ASN156 ILE157 VAL158 LEU168 ASP169	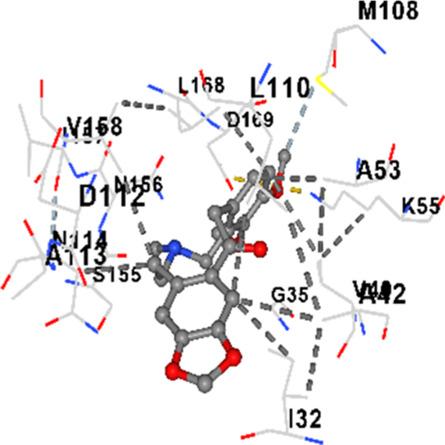
	P38	−8.4	Chain A: ARG5 PRO6 PHE8 PRO21 ARG23 TYR24 LYS45 ASP88 VAL89 PHE90 THR91 ALA93 ARG94 SER95 TYR342 VAL345 ILE346 SER347 PHE348 VAL349 PRO350 LEU353	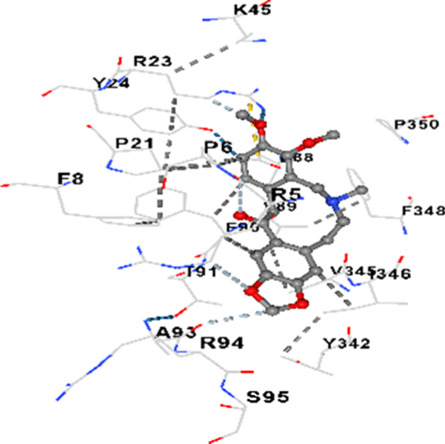
	AKT1	−9.8	Chain A: ARG15 GLY16 GLU17 TYR18 ASN53 ASN54 LEU78 GLN79 TRP80 THR81 THR82 ILE84 GLU85 THR87 LYS179 SER205 LEU210 THR211 LEU264 LYS268 VAL270 VAL271 TYR272 ARG273 ASP274 LYS276 THR291 ASP292 PHE293 GLY294 LEU295 CYS296 LYS297 CYS310 GLY311 THR312 TYR315 TYR326	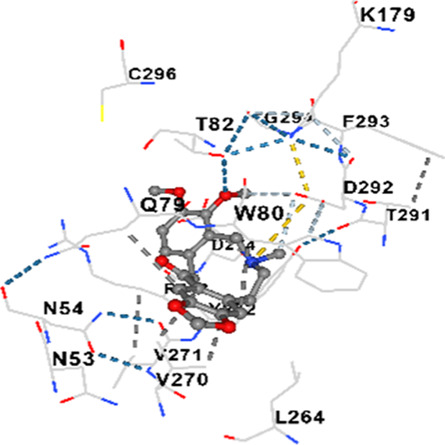
	GSK-3β	−8.2	Chain A: ILE62 GLY63 ASN64 GLY65 SER66 PHE67 GLY68 VAL70 ALA83 LYS85 VAL87 LEU88 GLN89 ASP90 ARG92 PHE93 LYS94 ASN95 ARG96 GLU97 VAL110 LEU132 ASP133 THR138 TYR140 ARG141 ARG180 ASP181 LYS183 GLN185 ASN186 LEU188 CYS199 ASP200 GLY202 SER203 ALA204 VAL214 TYR216 ILE217 Chain B: SER261 VAL263 ASP264 LYS292 PHE293 PRO294 GLN295 ILE296 LYS297	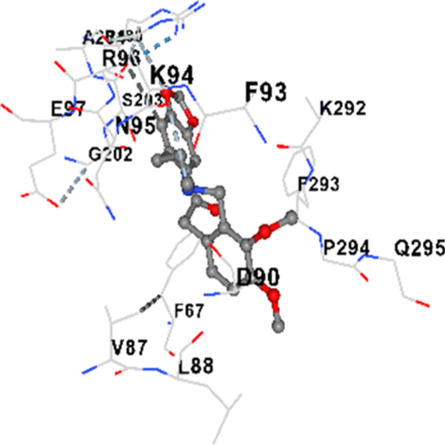
	TAU	−7.8	Chain L: ARG113 ALA116 THR119 VAL120 SER121 ILE122 PHE123 ASN143 ASP172 LYS174 ASP175 THR177 LYS212 SER213 PHE214 Chain H: PRO134 GLY135 SER136 ALA137 ALA138 GLN139 THR140 ASN141 SER142 MET143 VAL144 THR145 SER168 SER169 GLY170 VAL171 HIS172 THR190 SER193 TRP196 PRO197 PRO220	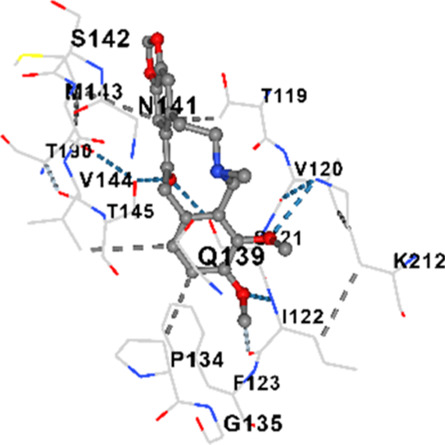
Tetrahydropalmatine	CACNA1C	−7.9	Chain A: LYS427 GLN428 GLU431 GLU432 LYS435 VAL585 SER586 LEU587 PHE588 PHE591 ASP592 VAL622 ARG623 LEU625 ARG626 LYS629 TRP634 ASN635 LEU637 SER638 ASN639 LEU640 VAL641 ALA642 LEU644 LEU645 GLN766 ILE1052 VAL1055 LEU1059 ILE1172 PHE1176 MET1177 ILE1180	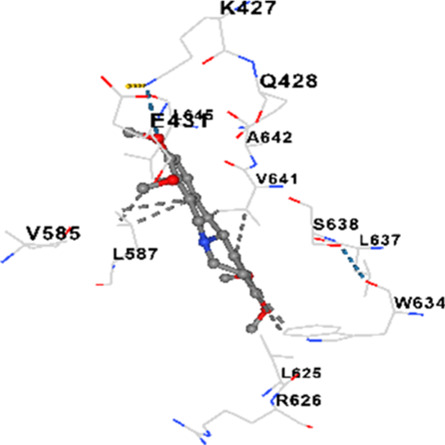
	CACNA1D	−8.7	Chain D: VAL312 ARG313 ASN448 PRO508 ASN509 TYR511 ASP516 PRO517 ASN518 TYR520 PRO525 ASN526 GLN528 ILE532 GLY533 VAL534 THR558 LEU559 ASP560 LEU562 ASP563 ALA564 GLU565 LEU566 GLU567 LYS571 ARG575 THR590 LEU591 VAL592 LYS593 SER594 GLN595 GLU597 ARG605 TYR607 ASP616 VAL622 GLU760 LYS765 LYS896 SER897 TYR898 TYR900 ILE975 GLN978 GLY1040 PRO1041 ARG1051 TYR1052 ARG1053 LYS1054	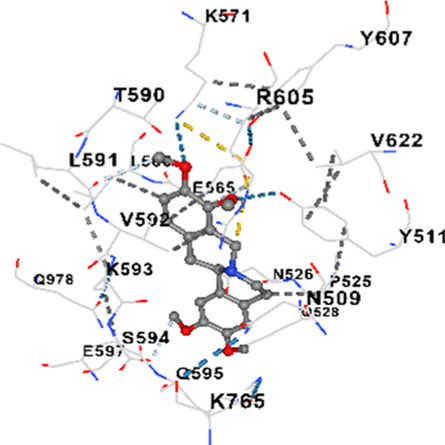
	ERK1/2	−8.2	Chain A: ILE31 GLY32 GLU33 GLY34 ALA35 TYR36 VAL39 ALA52 LYS54 ARG67 GLU71 ILE84 GLN105 ASP106 LEU107 MET108 GLU109 THR110 ASP111 TYR113 LYS114 LYS151 SER153 ASN154 LEU156 CYS166 ASP167 PHE168 GLY169 LEU170	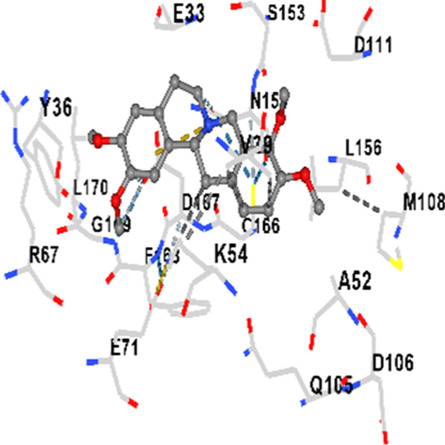
	JNK	−8.6	Chain B: LYS30 ILE32 GLY33 SER34 GLY35 ALA36 VAL40 ALA42 ALA53 LYS55 GLU73 MET77 ILE86 ILE106 MET108 GLU109 LEU110 MET111 ASP112 ALA113 ASN114 LYS153 SER155 ASN156 VAL158 LEU168 ASP169	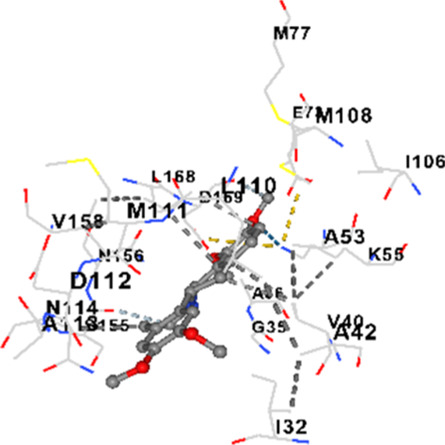
	P38	−7.1	Chain A: ARG5 PRO6 PHE8 PRO21 ARG23 TYR24 LYS45 THR46 ASP88 VAL89 PHE90 THR91 ALA93 ARG94 SER95 TYR342 VAL345 ILE346 PHE348 PRO350 LEU353	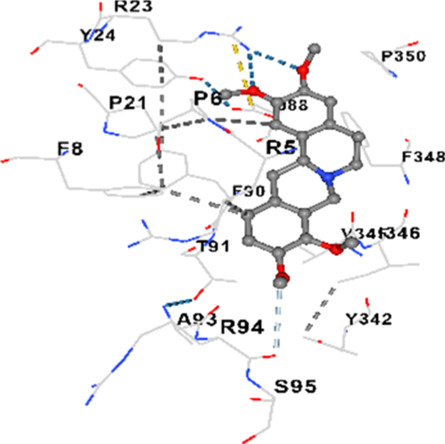
	AKT1	−9.4	Chain A: GLU17 TYR18 ASN53 ASN54 SER56 ALA58 GLN59 CYS77 LEU78 GLN79 TRP80 THR82 ILE84 GLU85 SER205 ARG206 HIS207 LEU210 THR211 ALA212 LEU213 TYR263 LEU264 LYS268 VAL270 VAL271 TYR272 ARG273 ASP274 ILE290 THR291 ASP292 GLY294 LEU295 CYS296 TYR326	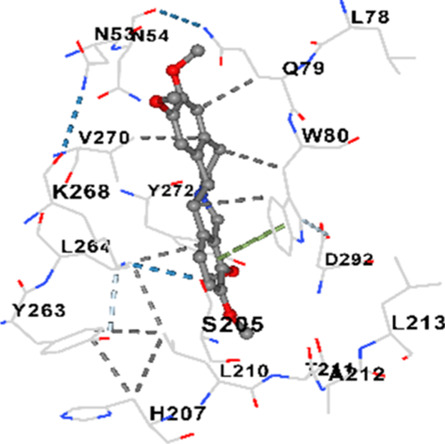
	GSK-3β	−7.9	Chain A: SER66 PHE67 LYS85 VAL87 LEU88 GLN89 ASP90 ARG92 PHE93 LYS94 ASN95 ARG96 GLU97 ARG180 PHE201 GLY202 SER203 ALA204 LYS205 PRO212 ASN213 VAL214 TYR216 ILE217 Chain B: VAL263 PHE291 LYS292 PHE293 PRO294 GLN295 ILE296 LYS297	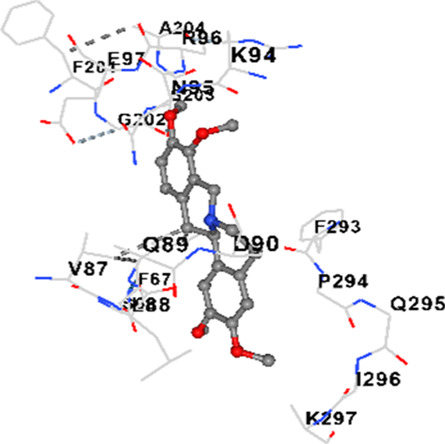
	TAU	−6.8	Chain L: ASP115 ALA116 ALA117 PRO118 THR119 VAL120 SER121 ILE122 PHE123 ASN142 ASN143 ASP172 LYS174 ASP175 THR177 LYS212 SER213 PHE214 Chain H: PRO134 GLY135 SER136 ALA137 ALA138 GLN139 THR140 ASN141 SER142 MET143 VAL144 THR145 SER169 GLY170 VAL171 HIS172 THR190 SER193 TRP196 PRO197 PRO220	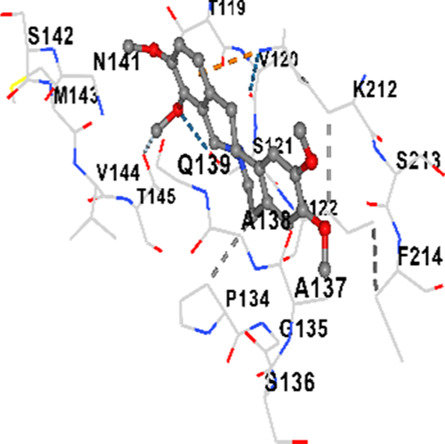
Tetrahydroberberine N-oxide	CACNA1C	−8.2	Chain A: LYS427 GLN428 GLU431 GLU432 LYS435 VAL585 SER586 LEU587 PHE588 PHE591 VAL622 ARG623 LEU625 ARG626 TRP634 LEU637 SER638 ASN639 VAL641 ALA642 LEU645 GLN766 VAL1055	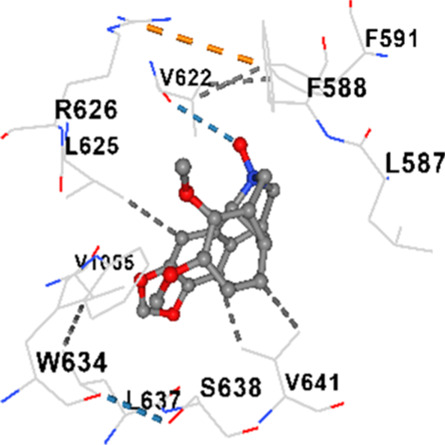
	CACNA1D	−7.6	Chain A: ARG1043 THR1045 ASP1046 GLU1047 ALA1048 LYS1049 SER1050 GLU1054 ARG1056 PHE1059 ILE1060 LEU1061 TYR1062 LYS1063 VAL1067 ARG1075 GLY1119 PRO1120 ILE1121 TYR1122 Chain D: ASP171 ILE172 TYR173 SER176 ILE178 VAL179 THR212 ARG239 ARG273 THR274 SER277 GLU278 GLU281 GLN391 HIS392 ARG396 GLU412 PRO414 SER415 ILE416 GLY417 ILE421	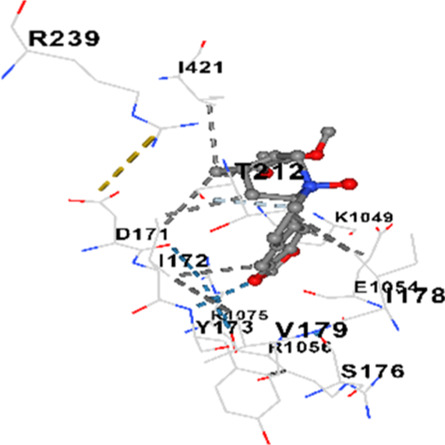
	ERK1/2	−7.8	Chain A: ILE31 GLY32 GLU33 GLY34 ALA35 TYR36 GLY37 MET38 VAL39 ALA52 LYS54 ARG67 GLU71 ILE84 GLN105 LEU107 MET108 THR110 ASP111 TYR113 LYS114 ASP149 LYS151 SER153 ASN154 LEU156 CYS166 ASP167 LEU170 VAL188 ALA189 THR190	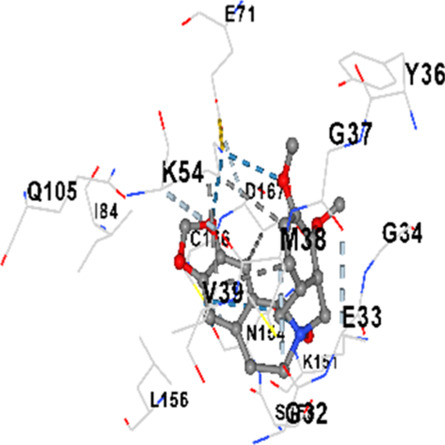
	JNK	−8.9	Chain B: LYS30 ILE32 GLY33 SER34 GLY35 ALA36 VAL40 ALA42 ALA53 LYS55 ILE86 MET108 GLU109 LEU110 MET111 ASP112 ALA113 ASN114 CYS116 GLN117 LYS153 SER155 ASN156 ILE157 VAL158 VAL159 LEU168 ASP169	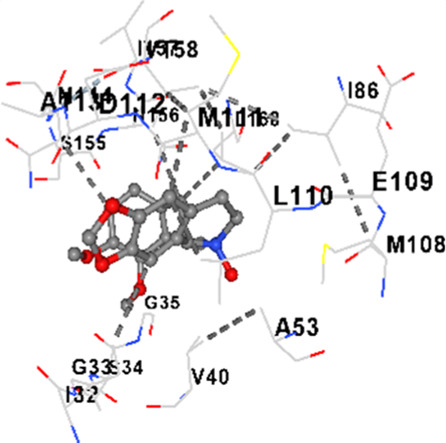
	P38	−7.3	Chain A: ARG5 PRO6 PHE8 PRO21 ARG23 LYS45 ASP88 VAL89 PHE90 THR91 ALA93 VAL345 ILE346 SER347 PHE348 VAL349 PRO350	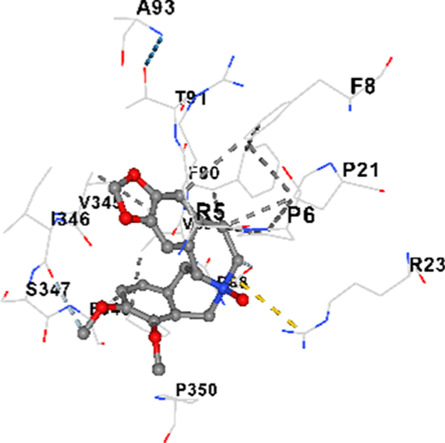
	AKT1	−6.9	Chain A: GLU17 TYR18 ASN53 ASN54 SER56 ALA58 GLN59 CYS60 CYS77 LEU78 GLN79 TRP80 THR82 ILE84 LYS154 LEU155 LEU156 GLY157 LYS158 VAL164 SER205 TYR229 GLU234 PHE236 LYS268 VAL270 VAL271 TYR272 ARG273 ASP274 LYS276 GLU278 ASN279 THR291 ASP292 PHE293 GLY294 LEU295 CYS296 LYS297 GLY311 THR312 TYR315	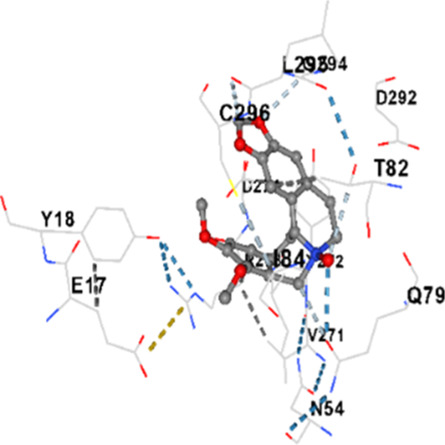
	GSK-3β	−8.0	Chain A: ILE62 GLY63 ASN64 GLY65 PHE67 VAL70 ALA83 LYS85 VAL87 LEU88 GLN89 ASP90 ASN95 ARG96 GLU97 GLN99 ILE100 LYS103 VAL110 LEU132 ASP133 TYR134 VAL135 PRO136 THR138 ARG141 ILE177 ARG180 LYS183 GLN185 ASN186 LEU188 CYS199 ASP200 GLY202 SER203 ALA204 LYS205 GLN206 TYR216 ILE217 Chain B: VAL263 PHE293 PRO294	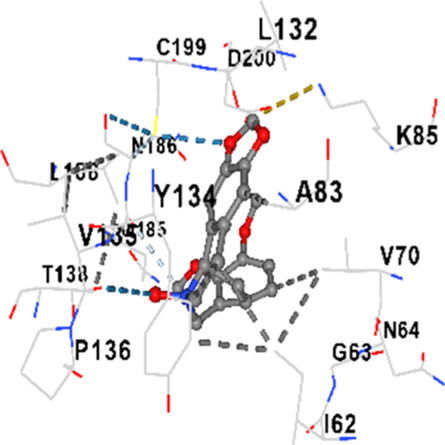
	TAU	−7.2	Chain L: THR119 PRO124 PRO125 SER126 SER127 GLU128 LEU130 ASN142 ASN143 TYR191 PHE214 ASN215 ARG216 GLU218 Chain H: PRO131 LEU132 ALA133 PRO134 GLY135 SER136 ALA137 MET143 THR145 HIS172 THR190 VAL219 PRO220	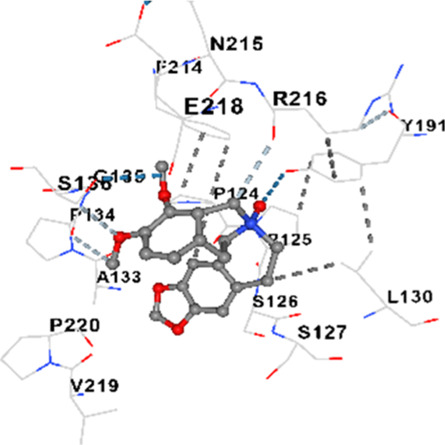

## 4 Discussion

NDs are neuronal diseases that progressively degenerate the structure and function of CNS or peripheral nervous system (PNS) and affect brain components (Ayeni et al., 2022). The most common NDs are Alzheimer’s disease (AD), Parkinson’s disease (PD), Huntington’s disease (HD), and amyotrophic lateral sclerosis (ALS) (Rekatsina et al., 2020). These diseases are a leading cause of death and affect a large number of people worldwide (Ayeni et al., 2022). Currently, although there are several drugs approved to treat NDs, the vast majority of them only help with the associated symptoms (Lamptey et al., 2022). Most NDs are known to have a multifactorial nature. Pathophysiologically, dysregulation of intracellular Ca^2+^ homeostasis, activation of MAPK family members ERK1/2, JNK, and p38 MAPK, and changes in specific proteins (Akt/GSK-3β/Tau) are the common features of the mechanisms of neurodegeneration (Abramov and Bachurin, 2021; Chen et al., 2009; Huang et al., 2017; Morén et al., 2022; Nimmrich and Eckert, 2013). According to this information, the discovery and/or development of natural multi-target drugs that simultaneously interact with the entire group of biotargets and processes involved in the pathogenesis of NDs seems quite promising (Abramov and Bachurin, 2021; Rekatsina et al., 2020).

Our previous results demonstrated that GGAE, which contain allocryptopine (major), tetrahydropalmatine, and tetrahydroberberine N-oxide suppressed oxidative stress (H_2_O_2_)-induced neuronal apoptosis, possibly by suppressing the mitochondrial apoptotic pathway and regulating the cell cycle in PC12 cells (Nigdelioğlu Dolanbay et al., 2021). Inflammation brought about by lipopolysaccharide (LPS) was suppressed by GGAE, which was attributed to their suppressor effects on the pro-inflammatory cytokines-mediators, and p38 MAPK in BV2 cells (Nigdelioğlu Dolanbay et al., 2023). Oxidative stress brought about by H_2_O_2_ was also suppressed by GGAE, which was attributed to their regulator effects on the NRF2-KEAP1 in PC12 cells (Nigdelioğlu Dolanbay et al., 2023).

In this article, experimental studies were conducted to discover plant-derived alkaloids with neuroprotective effects on the common mechanisms of NDs. The effects of GGAE obtained with chloroform solvent on dysregulation of intracellular Ca^2+^ homeostasis, activation of MAPK (ERK1/2, JNK and p38) signaling pathways, and dysregulation of Akt/GSK-3β/Tau neural death signaling pathway were investigated.

In our study, the suppressive effect of GGAE on [Ca^+2^]_i,_ which is increased by H_2_O_2_ in fPC12 cells, thus regulating Ca^+2^ homeostasis, was investigated. Various studies have shown that H_2_O_2_ causes cellular membrane depolarization and subsequently increases [Ca^+2^]_i_ through various ion channels (Ca^+2^ and Na^+^ channels) (Feng et al., 2013; Xiao et al., 2008). It has been shown that [Ca^+2^]_i_ increased ∼1.3-4.4 fold in PC12 cells treated with different damaging agents and durations compared with the control group (He et al., 2020; Xiao et al., 2008). Therefore, our results are consistent with the literature, and these studies provide evidence that the H_2_O_2_ agent and duration we treated to fPC12 cells are effective in increasing [Ca^+2^]_i_. It has been reported in the literature that alkaloids reduce the increased intracellular Ca^2+^ amount after oxidative stress, thus effectively protecting the cell (Ansari and Khodagholi, 2013; Xiao et al., 2008). In a study conducted by Xiao et al. (2008), an isoquinoline alkaloid, protopine, was used in an *in vitro* model of oxidative stress-induced damage in PC12 cells and H_2_O_2_, and it was reported that protopine directly blocked Ca^2+^ and Na^+^ channels, prevented Ca^2+^ influx, and inhibited PC12 cell apoptosis through Ca^2+^ antagonism. Similarly, our results showed that GGAE could protect fPC12 cells from oxidative damage by suppressing increased [Ca^2+^]_i_.

In our study, the inhibitory effect of GGAE on Cav_1.2_ and Cav_1.3_, which are L-type VGCC subtypes activated by H_2_O_2_ in fPC12 cells, was investigated at both gene and protein levels and accordingly, its potential as an L-type Ca^2+^ channel blocker was investigated. A study reported that H_2_O_2_ increased the cytosolic Ca^2+^ level in nucleus tractus solitarii (nTS) neurons via multiple VGCCs (Ostrowski et al., 2017). In two separate studies using hippocampal neurons treated with Aβ_25-35_ and creating a hypoxic environment in PC12 cells, Cav_1.2_ and Cav_1.3_ mRNA expression levels increased by 1.2–1.6 and 1.1-2.0 folds, respectively, compared with the control, while protein expression increased by ∼1.5–1.8 and 1.2-2.7 folds, respectively (Kim and Rhim, 2011; Li et al., 2015). These studies prove that the H_2_O_2_ we used in our study effectively activates L-type VGCCs. In the literature, it has been reported that various alkaloids (protopine and berberine) inhibit ion flow by directly blocking some ion channels, including Ca^+2^ channels (Sadeghnia et al., 2017; Xiao et al., 2008). In a study conducted by Sadeghnia et al. (2017), berberine alkaloid was shown to have a protective effect on glutamate-induced oxidative stress and neuronal apoptosis in PC12 and N2a neuronal cells, and this effect was associated with the L-type Ca^2+^ channel blocking effect of berberine. In a study conducted by Frangieh et al. (2022), rat pituitary GH3b6 cell line expressing Cav_1.2_ and Cav_1.3_ was used, and these L-type VGCC isoforms activated by different agents were inhibited by 3 different isoquinoline alkaloids (oxotephanin, thalmiculin and talifilin). Similarly, in our study, it was determined that the GGAE suppressed Cav_1.2_ and Cav_1.3_, which are L-type VGCC subtypes, at both the gene and protein levels. It was also noted in our study that this effect was parallel to each other at the gene and protein levels.

In our study, we investigated the effects of GGAE on reducing the increased p-ERK1/2/t-ERK1/2, p-JNK/t-JNK and p-p38/t-p38 protein ratio with H_2_O_2_ in fPC12 cells and thus inhibiting MAPK signaling pathways. Many studies in the literature have shown that H_2_O_2_-induced oxidative stress can trigger apoptosis through activation of MAPK pathways. In studies using different doses and durations of H_2_O_2_ with PC12 and SH-SY5Y cells, it was determined that p-ERK1/2, p-JNK, and p-p38 MAPK protein levels were increased by ∼1.3–4.4; 1.2-13, and 1.3 to 5 fold, respectively, compared with the control (Lee et al., 2017; Liu et al., 2014; Nakajima et al., 2009; Qi et al., 2018). Based on these findings, it can be suggested that 200 μM H_2_O_2_ for 24 h, as used in our study, is more effective in stimulating p-ERK and p-JNK activation compared with the studies conducted by Nakajima et al. (2009) and Qi et al. (2018), and in stimulating p38 MAPK activation compared with the study conducted by Nakajima et al. (2009). Studies have reported that some alkaloids obtained from plants inhibit p38 and ERK MAPK signaling pathways increased by amyloid beta (Aβ) in microglia cells, and inhibit the activation of p38 MAPK and ROS production in lipopolysaccharide (LPS)-induced microglia cells, thus suppressing neuroinflammation (Dang et al., 2023; Seo et al., 2018). In studies conducted with various plant extracts containing flavonoids, sapogenins, saponins and triterpenoids, it has been shown that the phosphorylation levels of ERK, JNK and p38 MAPK were suppressed ∼1.2-2.1-fold, 1.3-2.8-fold, and 1.3-3.9-folds, respectively, compared with the control (Lin et al., 2016; Liu et al., 2014; Lee et al., 2017). According to these studies, it can be suggested that the GGAE we used in our study was more effective on MAPK signaling pathway proteins activated as a result of treatment with the damaging agent, especially p-ERK and p-JNK, and showed higher suppression on p38 MAPK than that reported by Lee et al. (2017) for *Melandryum firmum* Rohrbach (Caryophyllaceae) extract. In our study, it was observed that GGAE suppressed the increase in phosphorylation levels of ERK, JNK, and p38 MAPK proteins caused by H_2_O_2_ agent, and reduced the p-ERK1/2/t-ERK1/2, p-JNK/t-JNK, and p-p38/t-p38 MAPK ratios. The mechanism underlying the inhibitory effect of GGAE on MAPK signaling pathways is thought to be the intracellular Ca^+2^ homeostasis and the blockade of L-type VGCC, which is quite remarkable.

In our study, we investigated the effect of GGAE on increasing the p-Akt/t-Akt protein ratio decreased by H_2_O_2_ in fPC12 cells and thus on the suppressive effects on neural death pathways. In the literature, it has been reported that the decrease in p-Akt levels with H_2_O_2_ is responsible for the increase in hyperphosphorylated Tau levels in AD (Kim et al., 2011; Pei et al., 2003). In studies using PC12 cells and different damaging agents, p-Akt protein levels were generally detected between ∼0.13 and 0.5 folds compared with the control (Huang et al., 2017; Jiang et al., 2016a; Jing et al., 2017; Lee et al., 2009; Xian et al., 2013). Based on these findings, it can be suggested that 24-h 200 μM H_2_O_2_ used in our study reduced p-Akt protein levels more effectively in fPC12 cells compared with the treatments performed with Aβ by Xian et al. (2013), with aluminum maltolate by Huang et al. (2017), and with 100 μM H_2_O_2_ by Lee et al. (2009). In our findings, the increase in p-Akt levels, especially in the 250 μg/mL group, is remarkable. This is important in terms of reducing hyperphosphorylated Tau levels in AH (Pei et al., 2003). This is because the increase in phosphorylation of Akt indicates that the GGAE we used may play a role in PI3K-mediated signaling pathways that are important in cell survival (Jing et al., 2017). Studies have reported that some alkaloids obtained from plants suppress the Akt signal increased by Aβ (Seo et al., 2018) and lead to the activation of PI3K/Akt, which inactivates GSK-3β through modulation of the phosphorylation status, which causes a decrease in p-APP and Aβ levels (Fan et al., 2019). In a study conducted by Xian et al. (2013), it was shown that the p-Akt protein level in fPC12 cells exposed to 20 μM Aβ_25−35_ for 24 h was suppressed from the control to ∼0.45. In the same study, it was determined that in groups where the commercial preparation (1–50 µM) of the isorynchophylline alkaloid found in *Uncaria rhynchophylla* (Miq.) Miq. ex Havil. was treated before Aβ_25−35_ treatment, the p-Akt protein level increased by ∼1.2-2.1 fold compared with the Aβ_25−35_-treated group. According to this study, it appears that the GGAE we used in our study is more effective in increasing the p-Akt level that was reduced as a result of treatment with the damaging agent. Therefore, it was observed that GGAE reversed the decrease in the phosphorylation level of Akt protein caused by H_2_O_2_ agent and increased the p-Akt/t-Akt ratio; thus, it was effective in protecting fPC12 cells against neural apoptosis. In some studies, it was reported that PI3K/Akt signaling pathway is effective in GSK-3β mediated Tau protein hyperphosphorylation and neural survival; inhibition of this pathway increased GSK-3β activity and induced Tau protein hyperphosphorylation (Baki et al., 2004; Lee et al., 2009). According to this information; the effect of our relevant active substance on the p-Akt pathway is also important in terms of suppressing the hyperphosphorylation of Tau protein associated with this pathway and thus ensuring microtubule stabilization.

In our study, the effect of GGAE on increasing the p-GSK-3β/t-GSK-3β protein ratio, which was decreased by H_2_O_2_ in fPC12 cells, was investigated. Our results showed that in H_2_O_2_-treated cells, p-GSK-3β protein level was suppressed from control to 0.42 (^#^ *p* < 0.05). In addition, it was determined that H_2_O_2_ or any experimental group did not cause a significant change in t-GSK-3β protein level compared with the control. In some studies conducted with PC12 cells, it has been reported that H_2_O_2_ significantly reduces the phosphorylation of GSK-3β (Lee et al., 2009; Liu et al., 2009). In the literature, in studies using PC12 cells and different damaging agents, the p-GSK-3β protein level was generally determined to be ∼0.25-0.6 folds compared with the control (Huang et al., 2017; Lee et al., 2009; Liu et al., 2009; Xian et al., 2013). Based on these findings, it can be suggested that 200 μM H_2_O_2_ for 24 h used in our study reduces phosphorylated protein levels more effectively in fPC12 cells compared with the treatments performed by Xian et al. (2013) with Aβ and Huang et al. (2017) with aluminum maltolate. In our findings, the increase in p-GSK-3β level, especially in the 250 μg/mL group, is remarkable. This is important for the prevention of GSK-3β-mediated Tau protein hyperphosphorylation and neuronal survival (Baki et al., 2004; Lee et al., 2009). In some studies, alkaloids have been shown to reduce the level of soluble and insoluble Aβ plaques in the brain by reducing GSK-3β activity and Tau hyperphosphorylation in HEK293 cells (Yu et al., 2011; Durairajan et al., 2012). In one study, it was shown that p-GSK-3β protein level in fPC12 cells exposed to 20 μM Aβ_25−35_ for 24 h was suppressed from control to ∼0.43. On the other hand, prior to Aβ_25−35_ treatment, treatment with *Uncaria rhynchophylla* (Miq.) Miq. ex Havil. was shown to increase the level of p-GSK-3β protein. In groups treated with the commercial preparation of isorhynchophylline (1–50 µM), a compound found in *U. rhynchophylla*, p-GSK-3β levels were increased by approximately 1.6–2.1 fold compared with the Aβ_25−35_-treated group (Xian et al., 2013). According to this study, it can be suggested that the GGAE is more effective in increasing the p-GSK-3β level, which was decreased as a result of treatment with the damaging agent. Therefore, it was determined that the GGAE reversed the decrease in the phosphorylation level of GSK-3β protein caused by the H_2_O_2_ agent in fPC12 cells and increased the p-GSK-3β/t-GSK-3β ratio. It is suggested that GGAE we used in our study protects the cells by increasing both p-Akt and p-GSK-3β levels.

In our study, we investigated the effects of GGAE on reducing the increased p-Tau (Ser 396)/Tau-5 and p-Tau (Thr 212)/Tau-5 protein ratio with H_2_O_2_ in fPC12 cells and thus suppressing neural death pathways. In the literature, it has been reported that oxidative stress is associated with Tau pathology and that cells overexpressing Tau protein have increased sensitivity to oxidative stress due to depletion of their peroxisomes (Zhao et al., 2013). In studies conducted with PC12 cells, there are also findings showing that ROS and toxins increase Tau phosphorylation and thus cause neurodegeneration (Li et al., 2015; Poppek et al., 2006). In studies conducted with different damaging agents using PC12 cells, it was generally determined that the p-Tau (Ser 396) protein level was between ∼1.25 and 2.25 folds compared with the control (Gu et al., 2018; Huang et al., 2017; Senavong et al., 2016; Wang et al., 2017). In studies conducted with different epitopes of another p-Tau protein, Thr, it was determined that the protein level was between ∼1.5 and 2.25 folds compared with the control (Senavong et al., 2016; Wang et al., 2017; Yao et al., 2016). Based on these findings, it can be suggested that 24-h treatment of 200 μM H_2_O_2_ used in our study increased the phosphorylated protein level in fPC12 cells more effectively compared with the treatments performed with different neurodegenerative agents by Gu et al. (2018) and Wang et al. (2017). In our findings, the decrease in p-Tau levels, especially in the 500 μg/mL group, is remarkable. This is important in terms of inhibiting apoptosis of neural cells and preventing Tau phosphorylation, especially in AD (Liu et al., 2009; Şahin et al., 2014). Studies have shown that various alkaloids eliminate effects such as insoluble and soluble Aβ levels and hyperphosphorylated Tau in the cortex and hippocampus of transgenic AD mice, and they also reduce Tau hyperphosphorylation in several *in vitro* systems (Durairajan et al., 2012; Liu et al., 2014; Yu et al., 2011). In one study, it was determined that p-Tau (Ser 396) protein level in fPC12 cells exposed to 20 μM Aβ_25−35_ for 24 h increased by ∼1.6-fold compared with the control, and p-Tau (Thr 205), a different epitope of p-Tau (Thr 212), increased by ∼1.4-fold compared with the control. In the same study using *Uncaria rhynchophylla* (Miq.) Miq. ex Havil it has been shown that rhynchophylline and isorynchophylline alkaloids (100 µM) of the species suppressed the p-Tau protein level (Ser 396) by ∼1.3 fold and the p-Tau (Thr 205) protein level by ∼1.2 and 1.4 fold, respectively (Xian et al., 2012). Accordingly; when the studies conducted with these two species from different families are compared, it is seen that GGAE are more effective than rhynchophylline and isorynchophylline alkaloids found in *Uncaria rhynchophylla* species. Therefore, it was determined that GGAE suppressed the increase in the phosphorylation level of Tau proteins formed by H_2_O_2_ agent in fPC12 cells and reduced the p-Tau/t-Tau ratio, thus having a neuroprotective effect against neural apoptosis. Studies have shown that phosphorylation of Tau proteins occurs via the Akt/GSK-3β signaling pathway, and that cell-damaging agents increase the phosphorylation of Tau proteins by decreasing p-Akt and p-GSK-3β protein levels (Gao et al., 2012; Huang et al., 2017; Jiang et al., 2016b; Xian et al., 2013). In line with this information, it has been determined that GGAE regulates the Akt/GSK-3β signaling pathway by increasing p-Akt and p-GSK-3β proteins and suppresses Tau hyperphosphorylation accordingly.

In our study, the potential interactions of alkaloids found in GGAE with various target proteins were analyzed by molecular docking method. In this context, the binding energies (vina scores) of alkaloids to target proteins and the amino acid residues they contact were determined. Molecular docking studies are a widely used *in silico* technique to estimate how well a ligand fits into the active site of a target protein, i.e., the binding strength and the stability of the complex. The binding energies obtained are expressed in kilocalories/mole (kcal/mol), and more negative scores mean stronger binding affinity and therefore a more stable ligand-protein interaction. In other words, the lower the value of the vina score, the higher specificity and strength the ligand holds onto the binding site of the protein, indicating that it may be a potential inhibitor or therapeutic agent (Baser et al., 2024; Şirin and Niğdelioğlu Dolanbay, 2024).

When the scores obtained within the scope of the study were evaluated, the order of the target proteins according to their binding energy to the ligands was as follows: JNK > AKT1 > ERK1/2 > CACNA1C > CACNA1D > GSK-3β > P38 > TAU. This order shows that there is a high binding potential between ligands and proteins that have critical roles in cellular signaling pathways, especially JNK and AKT1. On the other hand, the order of binding energy between ligands was as follows: Allocryptopine > tetrahydropalmatine > tetrahydroberberine N-oxide, and it was seen that allocryptopine was the compound that exhibited the lowest (i.e., the strongest) binding energy with all target proteins. This situation is supported by the fact that allocryptopine showed very low values ​​of −9.1 kcal/mol and −9.8 kcal/mol, respectively, in its interactions with JNK and AKT1.

In the literature, it is accepted that Vina scores below −6.0 kcal/mol indicate moderate binding affinity, and below −8.0 kcal/mol indicate strong binding affinity (Quiroga and Villarreal, 2016; Trott and Olson, 2010). In this context, the fact that many scores obtained in our study were below these threshold values ​​indicates that the compounds in question may be pharmacologically significant. This also reveals that the ligands in question may have a potential for biological activity and that it would be appropriate to confirm them with further experimental studies.

In addition, interaction types were analyzed during the visualization of protein-ligand complexes. Hydrogen bonds are important interactions that provide directional and specific binding between the protein and the ligand, and these bonds are shown with turquoise dotted lines. Electrostatic interactions express the attractive forces resulting from charge distribution differences and are represented with yellow dotted lines. Hydrophobic interactions, on the other hand, are interactions that occur between apolar regions and play an important role in the stability of the complex and are visualized with gray dotted lines (Baser et al., 2024; Şirin and Niğdelioğlu Dolanbay, 2024). Evaluating these interactions together has contributed to a more detailed understanding of the binding patterns and affinities of ligands to target proteins.

This study demonstrated that GGAE, which is rich in allocryptopine, tetrahydropalmatine, and tetrahydroberberine N-oxide, exhibits multi-target neuroprotective effects by modulating various cellular signaling pathways implicated in neurodegeneration. GGAE significantly suppressed the increase in intracellular Ca^2+^ ([Ca^2+^]i) levels induced by H_2_O_2_, thereby helping to restore calcium homeostasis. It also downregulated the gene and protein expression of Cav1.2 and Cav1.3, L-type voltage-gated calcium channel (VGCC) subtypes, indicating that GGAE may act as a natural L-type Ca^2+^ channel blocker.

Moreover, GGAE inhibited the phosphorylation of key proteins in the MAPK signaling pathways (p-ERK1/2, p-JNK, and p-p38) triggered by oxidative stress, thereby suppressing pro-apoptotic signaling and promoting cell survival. GGAE also enhanced the levels of p-Akt, which were reduced by H_2_O_2_, and increased the p-GSK-3β/t-GSK-3β ratio, suggesting that it downregulates GSK-3β activity and reinforces the neuroprotective Akt/GSK-3β signaling pathway. This regulatory effect is particularly important for preventing hyperphosphorylation of Tau proteins, which is a hallmark of AD pathology.

In line with this, GGAE significantly reduced the elevated levels of p-Tau (Ser396)/Tau-5 and p-Tau (Thr212)/Tau-5 caused by H_2_O_2_, highlighting its protective role against Tau-mediated neuronal damage.

Molecular docking (*in silico*) studies further supported these findings. Among the alkaloids tested, allocryptopine exhibited the strongest binding affinities, particularly with JNK (−9.1 kcal/mol) and AKT1 (−9.8 kcal/mol). Most of the binding energies obtained were below −8.0 kcal/mol, indicating high pharmacological potential. Visualization of protein-ligand interactions revealed key hydrogen bonds, electrostatic forces, and hydrophobic interactions contributing to the stability and specificity of these complexes. Altogether, these results suggest that GGAE holds strong promise as a natural, multi-target therapeutic candidate for the treatment of NDs.

The limitations of the study are as follows: The findings of this study demonstrate that GGAE possesses significant therapeutic potential against neurodegenerative diseases. However, several limitations should be considered. Firstly, the study was conducted solely on the PC12 cell line, an *in vitro* model that does not fully replicate the complexity of the human central nervous system. Therefore, the direct applicability of the results to *in vivo* conditions remains limited. In addition, the neuroprotective effects of GGAE were not validated in animal models, which are essential for assessing biological efficacy and safety in a physiological context. The extract contains multiple alkaloids, and while the study focused on three major compounds, the roles of minor constituents and their potential synergistic or antagonistic effects were not fully explored. A detailed dose–response analysis was not included, making it difficult to determine the minimum effective dose or toxic thresholds. The experiments were based on short-term exposure; hence, the long-term or chronic effects of GGAE remain unknown. Furthermore, the pharmacokinetic properties and bioavailability of the alkaloids were not assessed, which are crucial parameters for drug development. Although molecular docking revealed strong binding affinities to target proteins, these interactions were not experimentally validated for specificity, raising the possibility of off-target effects. Finally, the study did not compare the neuroprotective efficacy of GGAE with existing standard treatments, limiting the contextual understanding of its therapeutic value. These limitations highlight the need for further in-depth studies to validate and expand upon the promising results observed.

## 5 Conclusion

In conclusion, this study has shown that the GGAE provides intracellular Ca^+2^ homeostasis, is an L-type Ca^+2^ channel blocker, dephosphorylates p-ERK1/2, p-JNK and p-p38 proteins, phosphorylates p-Akt (Ser 473) and p-GSK-3β (Ser 9) proteins, dephosphorylates p-Tau (Ser 396) and p-Tau (Thr 212) proteins and interacts with each other. Also, alkaloids (allocryptopine, tetrahydropalmatine, and tetrahydroberberine N-oxide) found in the extract exhibit strong binding energies to target proteins (AKT1, CACNA1C, CACNA1D,ERK1/2, GSK-3β, JNK, P38, and TAU).

## Data Availability

The raw data supporting the conclusions of this article will be made available by the authors, without undue reservation.
