# Supplementary material for: Allocryptopine, tetrahydropalmatine, and tetrahydroberberine N-oxide alkaloids alleviate cellular stress by modulating calcium homeostasis and the MAPK and akt/GSK-3β/tau signaling pathways
Source: Front Pharmacol. 2025 Nov 25;16:1589390. doi: 10.3389/fphar.2025.1589390 (PMC12685886; doi:10.3389/fphar.2025.1589390)
Supplement: Supplementary file 1 [file DataSheet1.zip › Supplementary Materials/Graphical abstract_Publication license.pdf]

49 Spadina Ave. Suite 200
Toronto ON M5V 2J1 Canada
www.biorender.com

Confirmation of Publication and Licensing Rights

September 7th, 2024

Subscription:	*Student Plan - Academic*
Agreement number:	*KU27A0G3P7*
Publication name:	*Molecular Neurobiology*
Citation to Use:	*Created in BioRender. æ— in, S. (2024) BioRender.com/h17x406*

To whom this may concern,

This document is to confirm that Seda Şirin has been granted a license to use the BioRender Content, including icons, templates, and other original artwork, appearing in the attached Completed Graphic pursuant to BioRender's Academic License Terms. This license permits BioRender Content to be sublicensed for use in publications (journals, textbooks, websites, etc.).

All rights and ownership of BioRender Content are reserved by BioRender. All Completed Graphics must be accompanied by the following citation: "Created in BioRender. Şirin, S. (2024) BioRender.com/h17x406 ".

BioRender Content included in the Completed Graphic is not licensed for any commercial uses beyond use in a publication. For any commercial use of this figure, users may, if allowed, recreate it in BioRender under an Industry BioRender Plan.

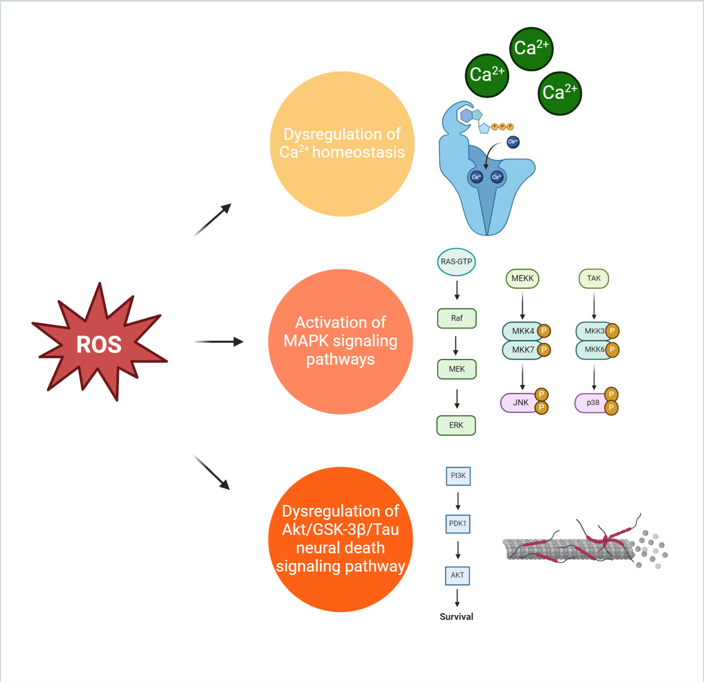

For any questions regarding this document, or other questions about publishing with BioRender, please refer to our BioRender Publication Guide, or contact BioRender Support at support@biorender.com.
