# Supplementary figures and images for: Allocryptopine, tetrahydropalmatine, and tetrahydroberberine N-oxide alkaloids alleviate cellular stress by modulating calcium homeostasis and the MAPK and akt/GSK-3β/tau signaling pathways
Source: Front Pharmacol. 2025 Nov 25;16:1589390. doi: 10.3389/fphar.2025.1589390 (PMC12685886; doi:10.3389/fphar.2025.1589390)

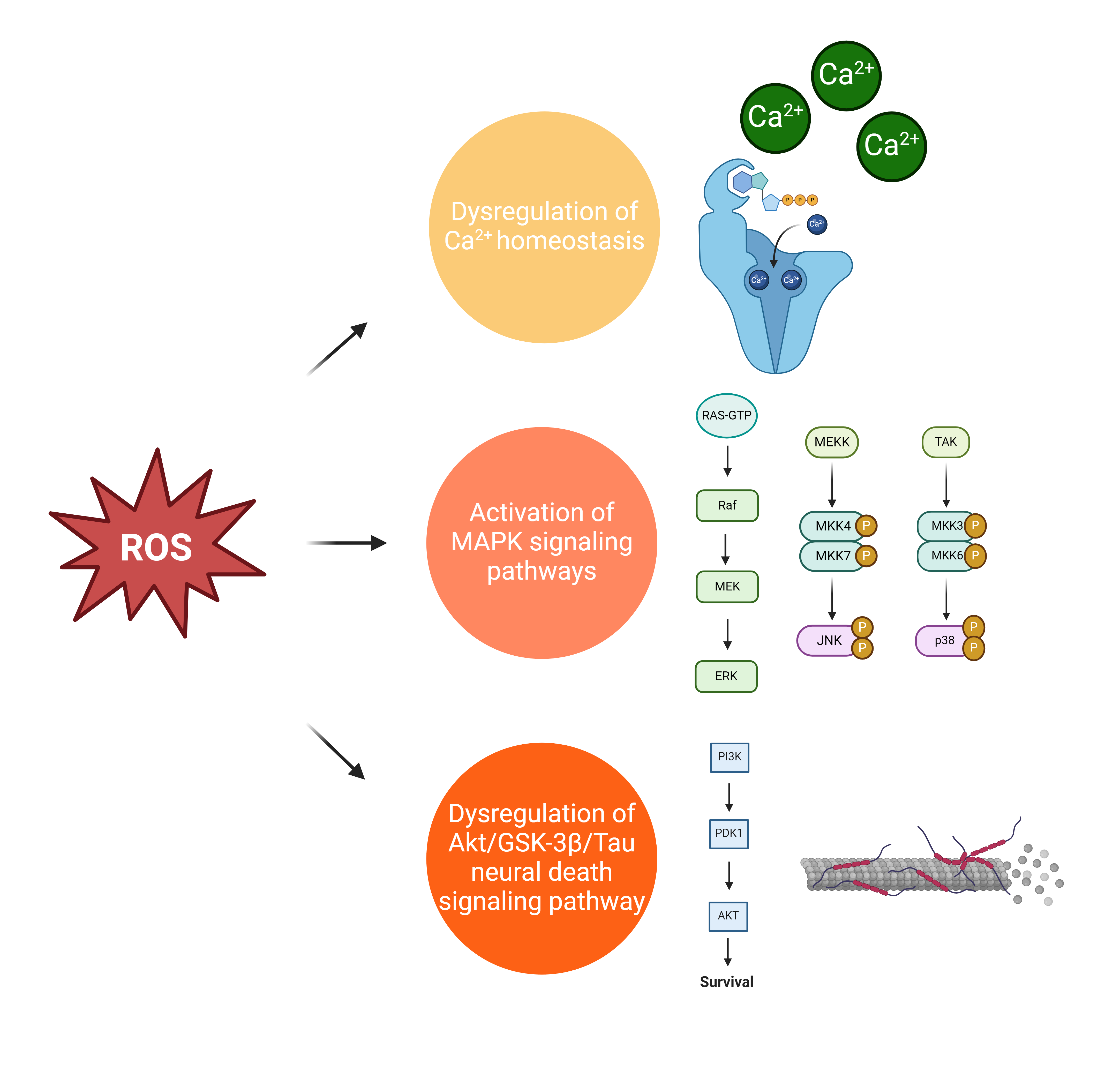

Supplement: Supplementary file 1 [file DataSheet1.zip › Supplementary Materials/Graphical abstract.png]

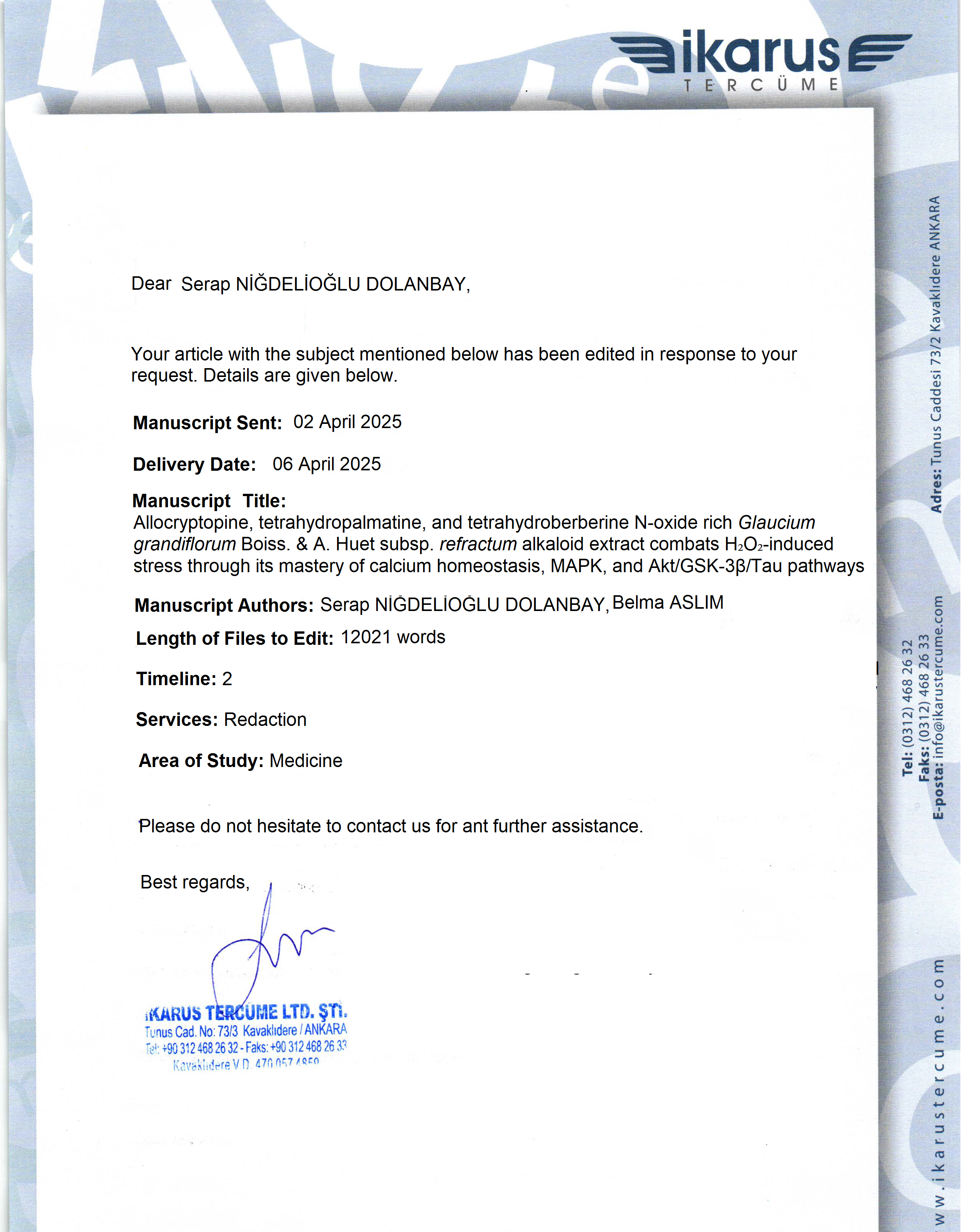

Supplement: Supplementary file 1 [file DataSheet1.zip › Supplementary Materials/Language edit certificate.tif]

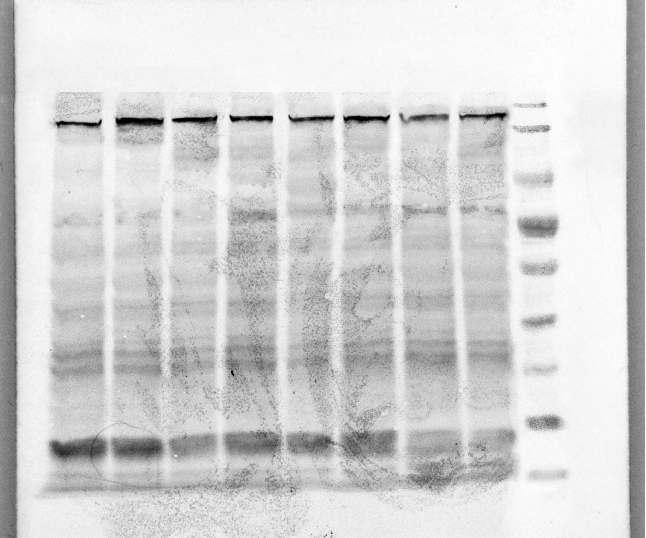

Supplement: Supplementary file 1 [file DataSheet1.zip › Supplementary Materials/Western blot membranes/cav1.2.jpg]

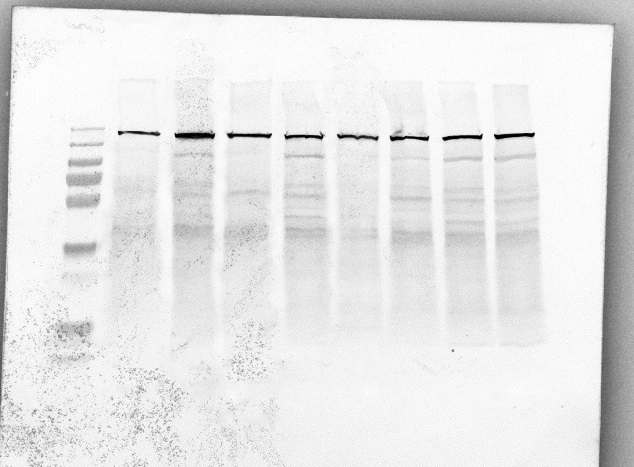

Supplement: Supplementary file 1 [file DataSheet1.zip › Supplementary Materials/Western blot membranes/cav1.3.jpg]

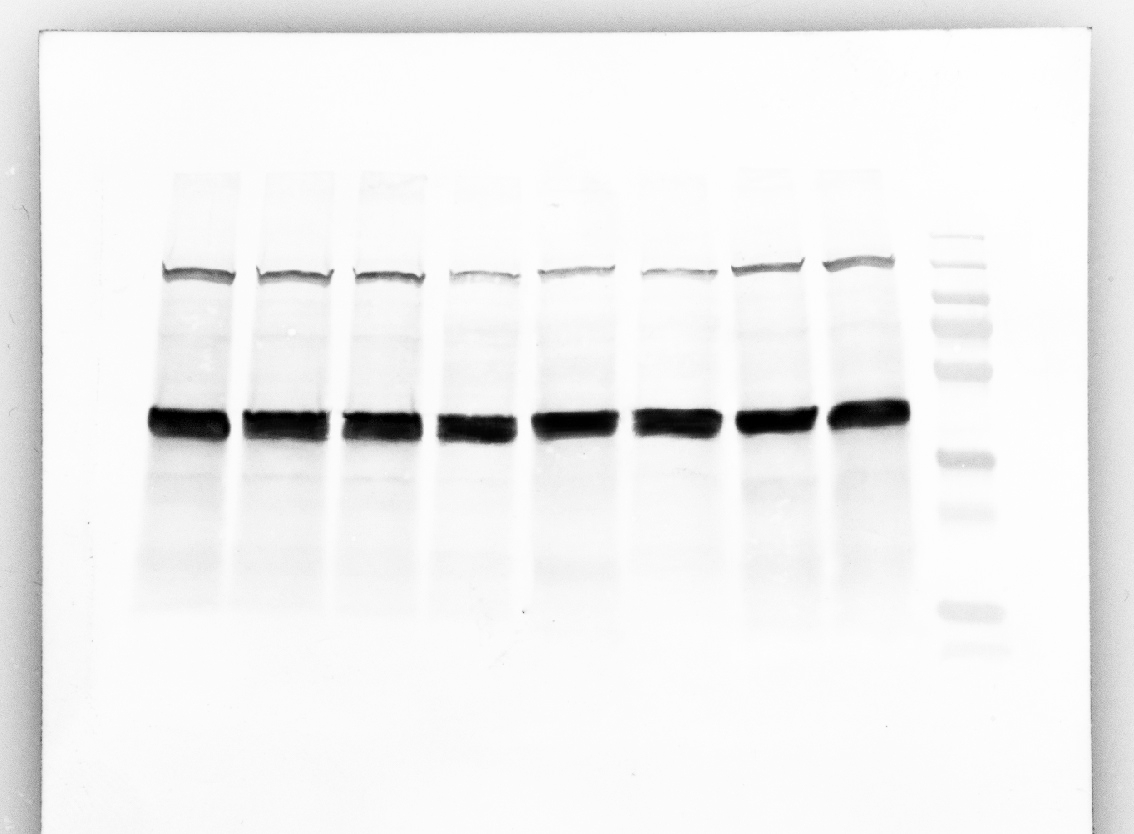

Supplement: Supplementary file 1 [file DataSheet1.zip › Supplementary Materials/Western blot membranes/gapdh.jpg]

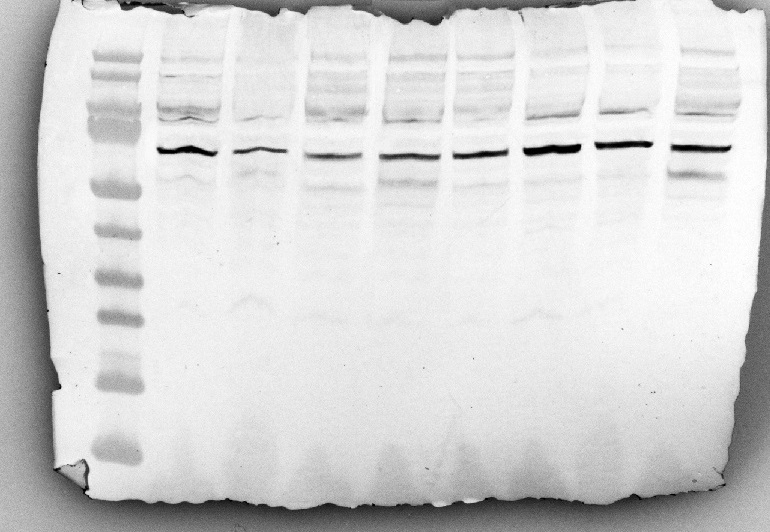

Supplement: Supplementary file 1 [file DataSheet1.zip › Supplementary Materials/Western blot membranes/p-akt.jpg]

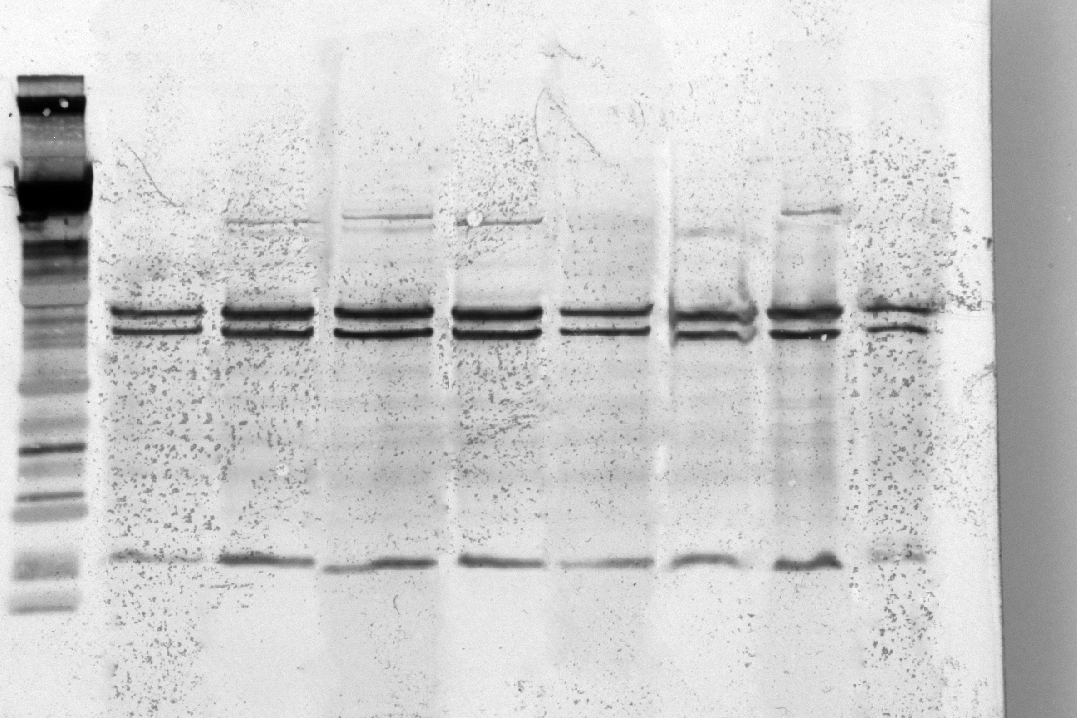

Supplement: Supplementary file 1 [file DataSheet1.zip › Supplementary Materials/Western blot membranes/p-erk.jpg]

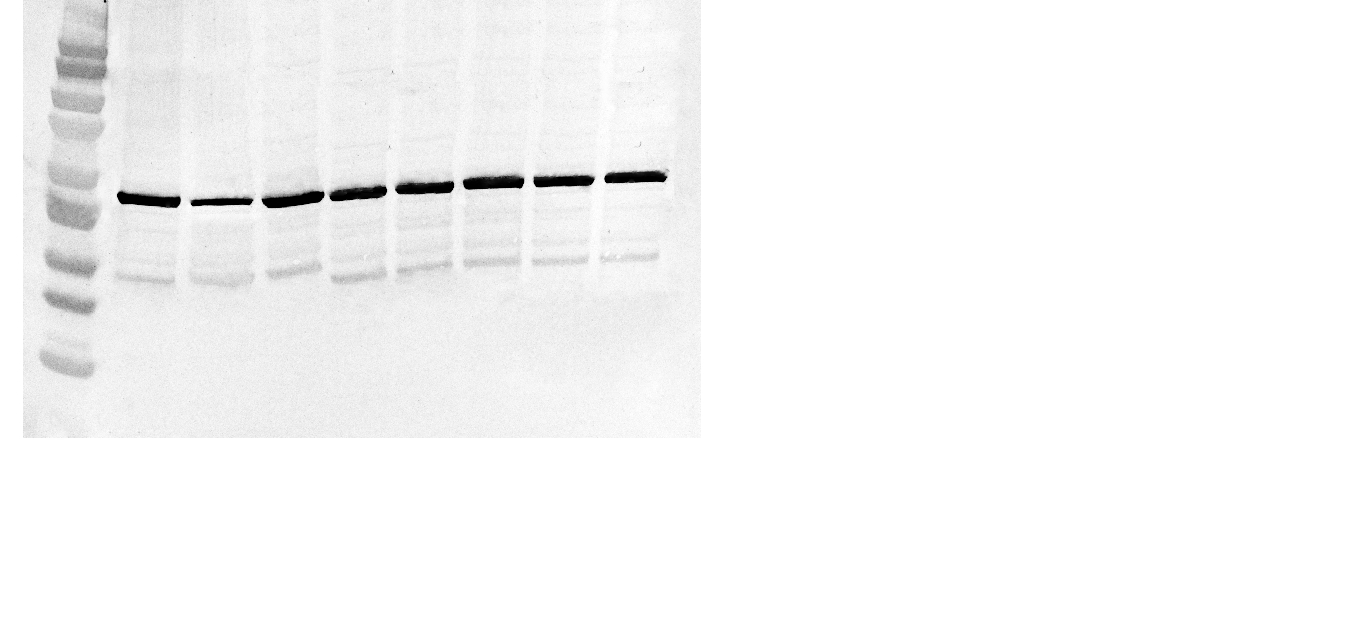

Supplement: Supplementary file 1 [file DataSheet1.zip › Supplementary Materials/Western blot membranes/p-gsk.jpg]

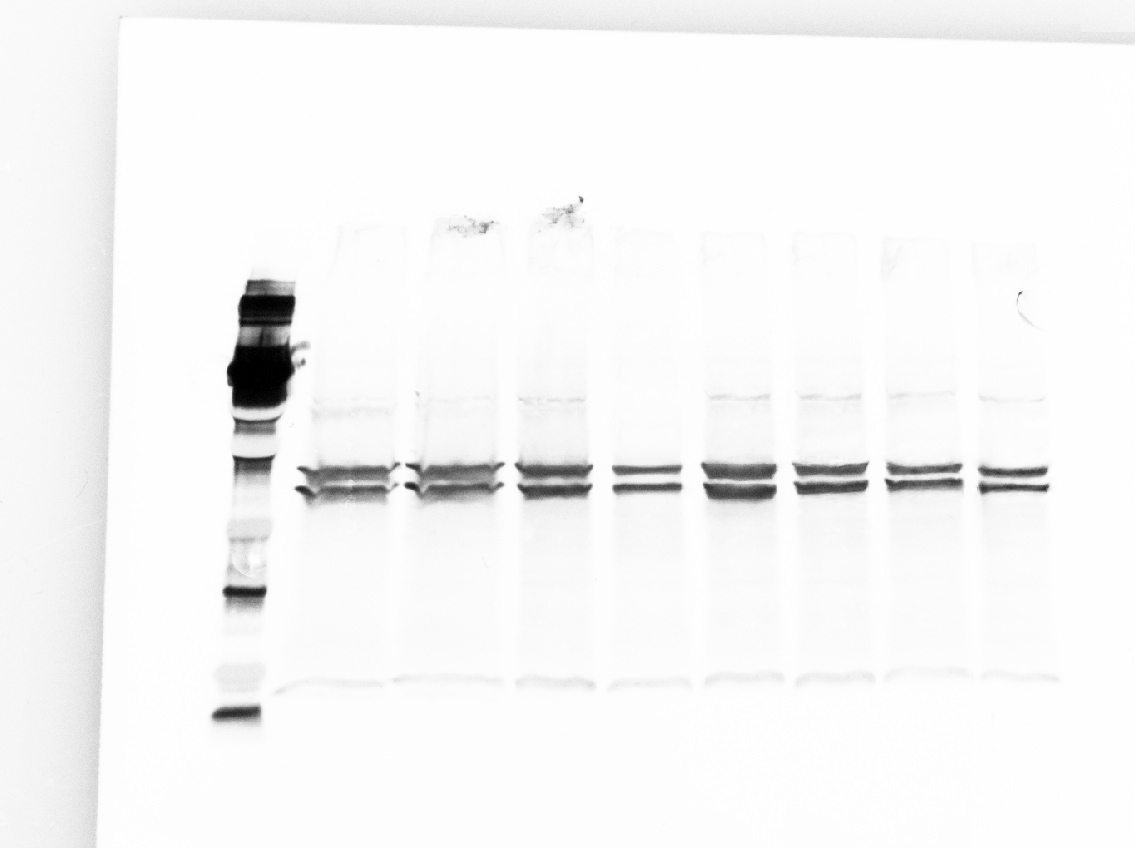

Supplement: Supplementary file 1 [file DataSheet1.zip › Supplementary Materials/Western blot membranes/p-jnk.jpg]

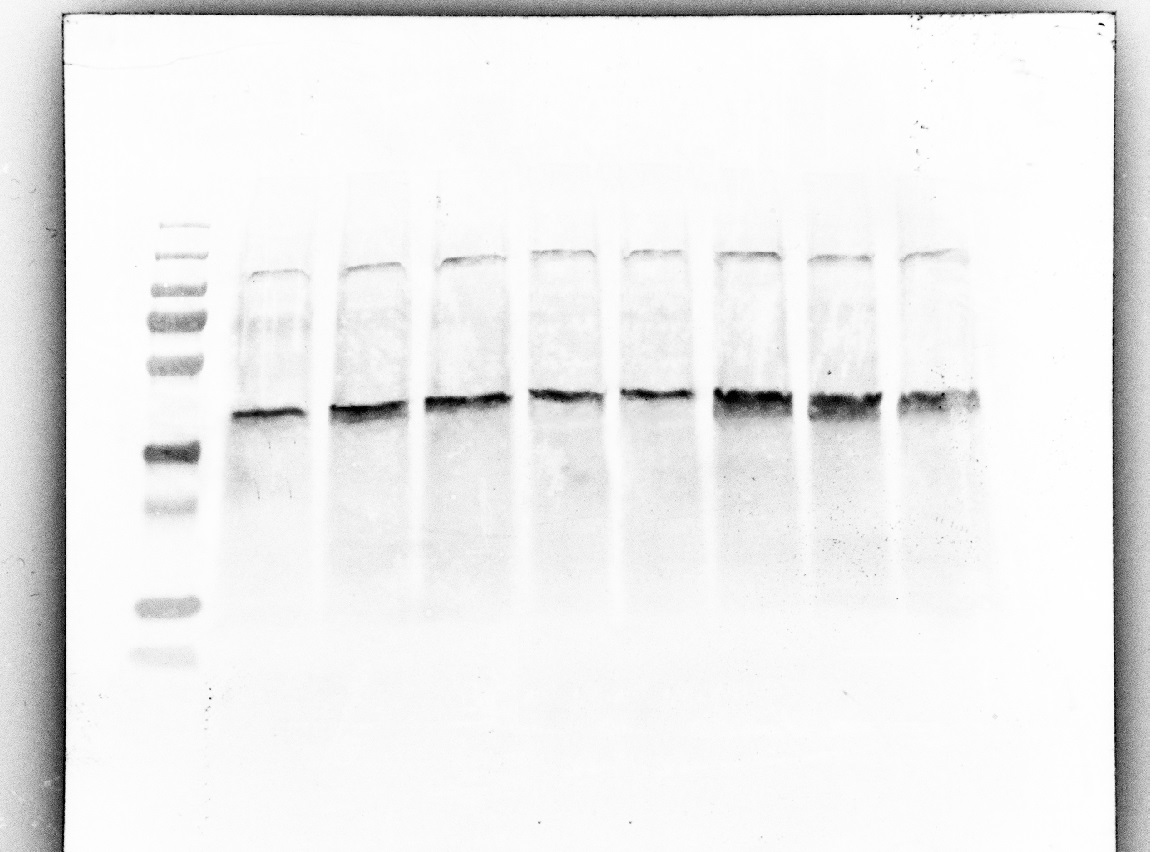

Supplement: Supplementary file 1 [file DataSheet1.zip › Supplementary Materials/Western blot membranes/p-p38.jpg]

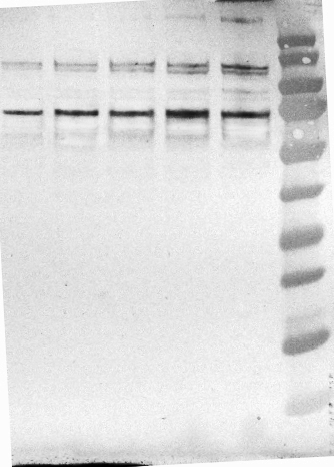

Supplement: Supplementary file 1 [file DataSheet1.zip › Supplementary Materials/Western blot membranes/ser396.jpg]

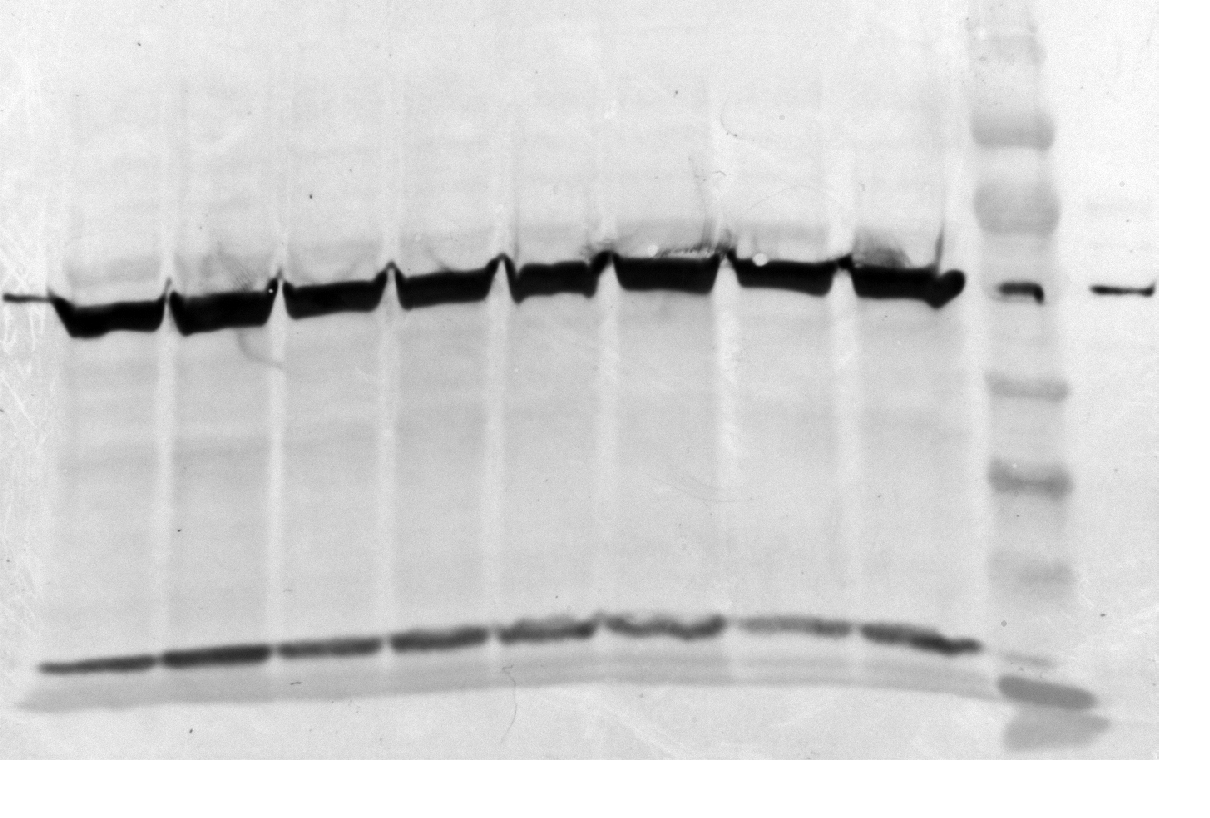

Supplement: Supplementary file 1 [file DataSheet1.zip › Supplementary Materials/Western blot membranes/t-akt.png]

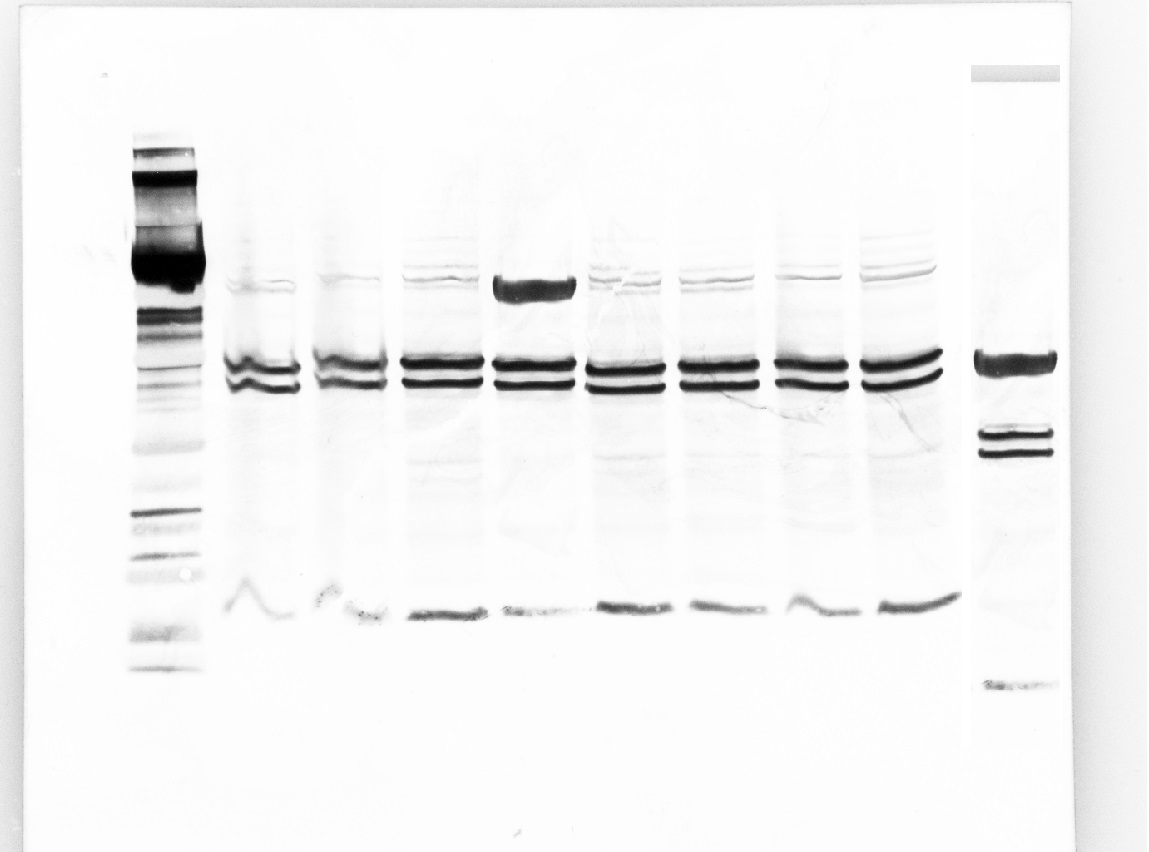

Supplement: Supplementary file 1 [file DataSheet1.zip › Supplementary Materials/Western blot membranes/t-erk.jpg]

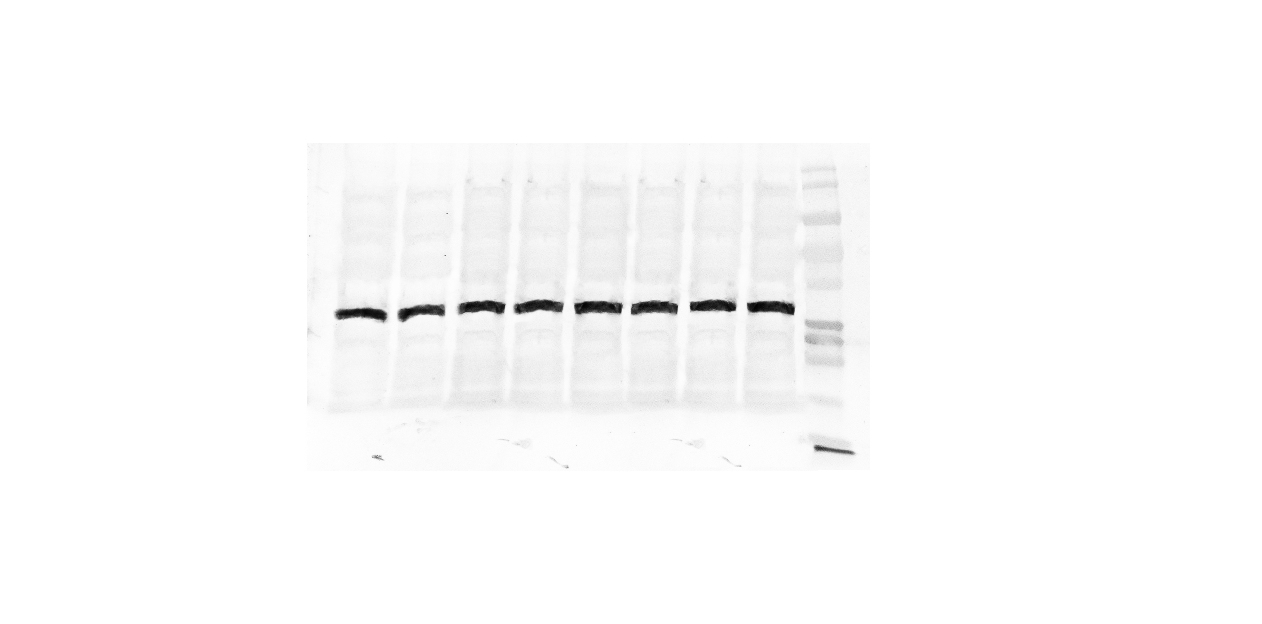

Supplement: Supplementary file 1 [file DataSheet1.zip › Supplementary Materials/Western blot membranes/t-gsk.jpg]

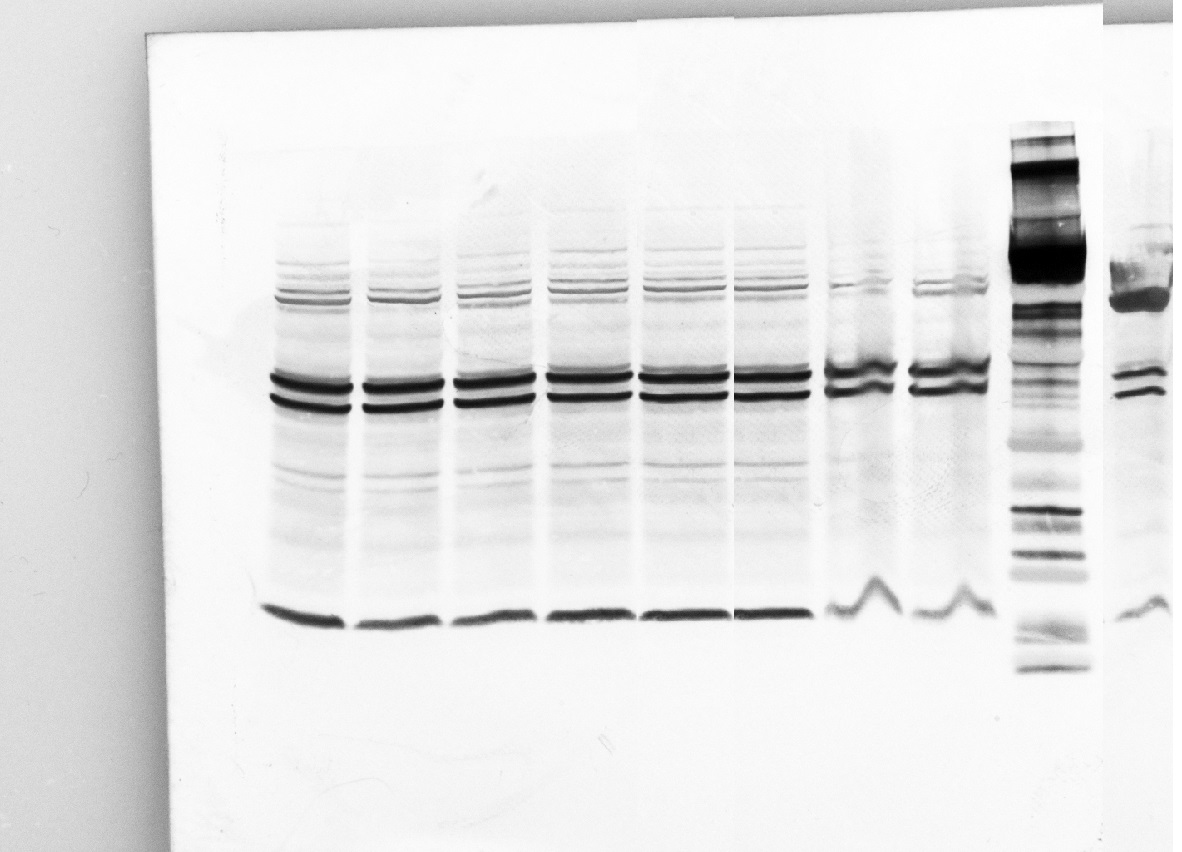

Supplement: Supplementary file 1 [file DataSheet1.zip › Supplementary Materials/Western blot membranes/t-jnk.jpg]

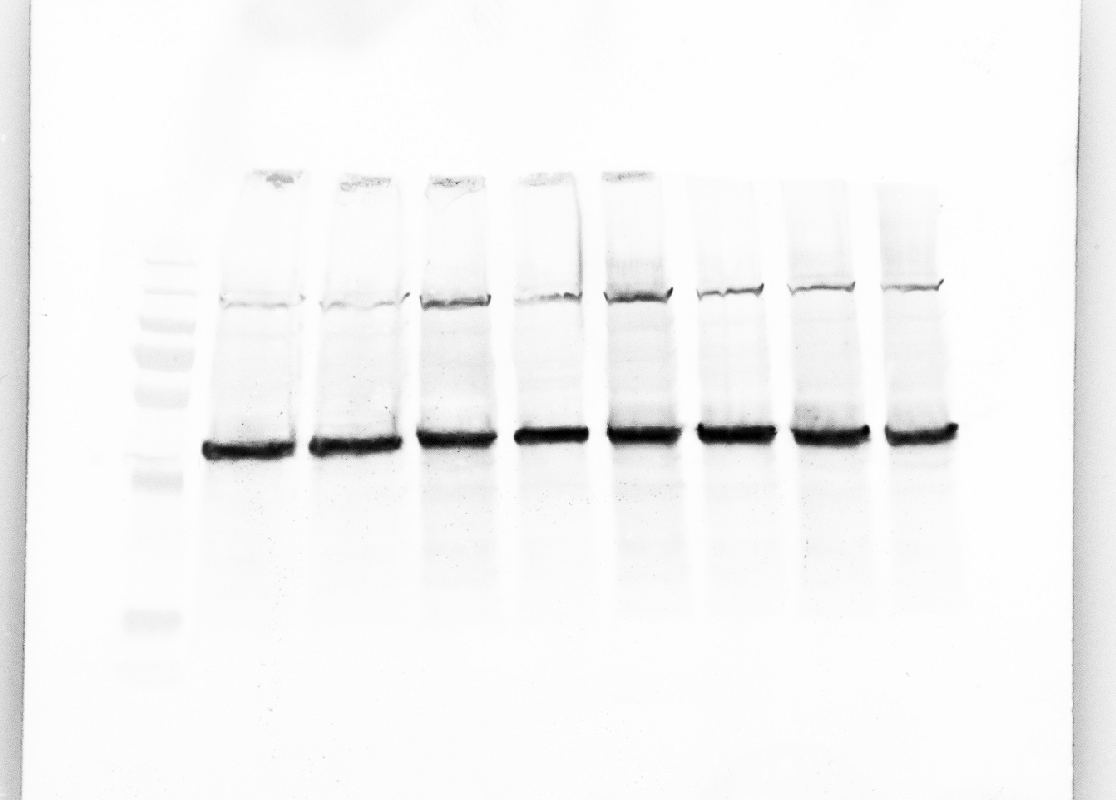

Supplement: Supplementary file 1 [file DataSheet1.zip › Supplementary Materials/Western blot membranes/t-p38.jpg]

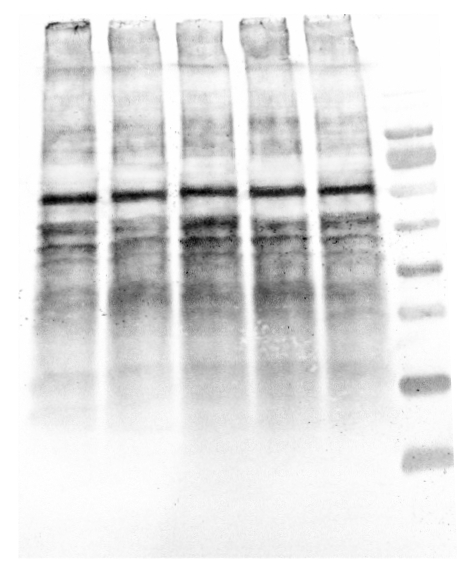

Supplement: Supplementary file 1 [file DataSheet1.zip › Supplementary Materials/Western blot membranes/tau5.jpg]

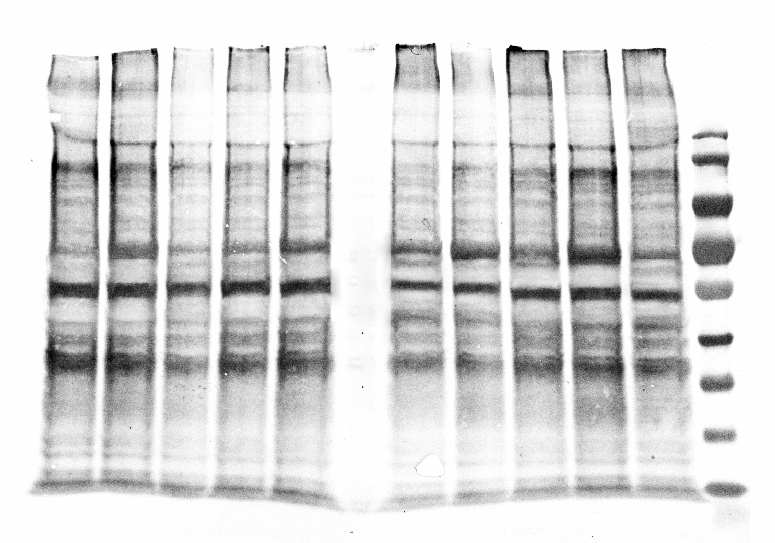

Supplement: Supplementary file 1 [file DataSheet1.zip › Supplementary Materials/Western blot membranes/thr212.jpg]
